# Identification of QTLs for behavioral reactivity to social separation and humans in sheep using the OvineSNP50 BeadChip

**DOI:** 10.1186/1471-2164-15-778

**Published:** 2014-09-09

**Authors:** Dominique Hazard, Carole Moreno, Didier Foulquié, Eric Delval, Dominique François, Jacques Bouix, Guillaume Sallé, Alain Boissy

**Affiliations:** INRA, UMR1388 Génétique, Physiologie et Systèmes d’Elevage, F-31326 Castanet-Tolosan, France; Université de Toulouse INPT ENSAT, UMR1388 Génétique, Physiologie et Systèmes d’Elevage, F-31326 Castanet-Tolosan, France; Université de Toulouse INPT ENVT, UMR1388 Génétique, Physiologie et Systèmes d’Elevage, F-31076 Toulouse, France; INRA UE321 La Fage, Saint Jean et Saint Paul, F-12250 Roquefort-sur-Soulzon, France; INRA UMR1213 Herbivores, F-63122 Saint-Gènes-Champanelle, France; Université de Clermont, VetAgro Sup, UMR1213 Herbivores, BP 10448, F-63000 Clermont-Fd, France; Université de Lyon, VetAgro Sup, UMR1213 Herbivores, F-69280 Marcy l’Etoile, France

**Keywords:** Sheep, Behavioral reactivity, Quantitative trait loci, Gregariousness, Human, Cortisol

## Abstract

**Background:**

Current trends in sheep farming practices rely on animals with a greater level of behavioral autonomy than before, a phenotype that actively contributes to the sustainability of animal production. Social reactivity and reactivity to humans are relevant behavioral traits in sheep, known for their strong gregariousness and weak tolerance to handling, which have previously been reported with moderate to high heritabilities. To identify loci underlying such behaviors, we performed a genome study in Romane lambs.

**Results:**

The experiment was carried out on 934 male and female lambs allocated into 9 half-sib families (average of 103 lambs per family) and reared outside. After weaning, all the lambs were individually exposed to 4 standardized behavioral tests combining social isolation, exposure to humans or handling, confinement and novelty (i.e. arena test, corridor test, isolation box test, shearing test). A broad range of behaviors including vocalizations, locomotion, vigilance and flight distance, as well as the cortisol response to handling, were collected. All lambs were genotyped using the Illumina OvineSNP50 BeadChip. QTL detection was performed by linkage, association and joint linkage and association analyses using the QTLmap software. Five main QTL regions were identified on sheep chromosomes (*Ovis Aries* Region, OAR) 12, 16, 19, 21 and 23 among many other QTLs with small to moderate effects. The QTLs on OAR12, 16 and 21 showed significant associations with social reactivity. The QTLs on OAR19 and 23 were found to be associated with reactivity to humans. No overlapping QTLs were identified for the different traits measured in the behavioral tests, supporting the hypothesis that different genetic factors influence social reactivity and tolerance to humans.

**Conclusion:**

The results of this study using ovine SNP data suggest that in domestic sheep the behavioral responses to social separation and exposure to humans are under polygenic influence. The most relevant QTLs reported in the present study contain interesting candidate genes previously described to be associated with various emotional and social behaviors in mammals.

**Electronic supplementary material:**

The online version of this article (doi:10.1186/1471-2164-15-778) contains supplementary material, which is available to authorized users.

## Background

Behavioral adaptation of farm animals to various rearing conditions is a growing concern. Whatever the rearing system, farms animals are often faced with physical and social environmental changes that they have to cope with by developing adapted behavioral and/or physiological responses [1–3]. Animals respond, both behaviorally and physiologically, to challenges in order to maintain homeostasis [4, 5] that contributes highly to their fitness. The development of modern production systems and selection for production traits has induced a rise in the prevalence of behavioral disorders [6, 7]. The difficulties experienced by some animals when faced with social challenges, such as isolation or social instability in sheep [8], may cause poor welfare and production loss [9–14]. On the other hand, the reduced manning levels on livestock farms combined with the increased size of herds leads to a lesser exposure to humans [15]. As a consequence, animals are more likely to experience stress during routine husbandry procedures [16]. Human-animal interaction studies previously reported the negative effects of high behavioral reactivity to humans on production traits [15, 16]. The social skills of the farm animal, including social tolerance, social facilitation or maternal behavior, are actively involved in helping it adapt to its environment [17].

Genetic selection for behavioral traits could be an advantageous strategy aimed at improving the ability of animals to adapt to modern rearing conditions by reducing, for instance, the susceptibility of an animal to changes in its physical environment, limiting excessive fear of humans and improving sociability [18]. In the past, most studies on the genetics of behavior in mammals have focused on rodents, where the existence of inbred lines, for instance in mice, has increased the power to detect quantitative trait loci (QTL) [19] and to identify candidates genes related to behavior [20–22]. Although findings in rodents may give new insights into the understanding of various behaviors in mammals, some gene effects (i.e. allelic variants) may be species-specific, and therefore QTLs identified in rodents may not be relevant in livestock species, and *vice versa*. Moreover, the exploration of some behaviors, such as reactivity to humans, is only relevant in livestock species.

Animal behavior is a complex concept that has been shown to be influenced by a large variety of factors. A number of studies have focused on the development of standard tests to evaluate the variability of the behavioral response of sheep to novelty, social isolation, attraction for conspecifics or the presence of a human [23–26]. For instance, the arena test has been used in sheep to evaluate temperament [27], to assess sociability [28] and to investigate a possible selection criterion for lamb-rearing ability [14, 29]. The arena test has been combined with an isolation box test to divergently select for temperament in sheep [30, 31].

In livestock species, and more particularly in sheep, genetic variation between breeds has been reported to explain differences of behavioral reactivity in standard tests [25, 32–34]. Medium to high heritabilities (0.20 to 0.49) have been reported within various breeds of sheep for several behavioral traits involved in social reactivity [25, 35]. As regards to the reactivity to humans in particular, heritability estimates were moderate both in sheep (0.17 to 0.32) [25] and cattle (0.17 to 0.24) [12, 13, 36]. However, QTL studies for behavior are still scarce in livestock species (for review see [37]). For instance, QTLs for social behavior have been found in fish [38] and chickens [39], and QTLs for reactivity to humans have been reported in dairy and beef cattle [40, 41].

In sheep, while major genes or QTLs have been reported for reproduction [42], production [43] and health traits [44] (for a review see [45]), no such studies have been undertaken for behavioral traits. The recent development of genome-wide analyses in sheep provides new opportunities for better understanding of the genetic components of various behaviors, with potential applications in the improvement of such traits. Taking advantage of such developments and considering the existing genetic variability for some behavioral traits found in domestic sheep, the aim of the present study was to perform QTL detection for behavioral and physiological traits in purebred sheep. We focused our study in particular on the reactivity of sheep measured in response to social isolation and/or human presence/handling.

## Methods

### Animals and management

The experimental animals were Romane lambs (historically named INRA401), a fixed crossbreed between Romanov x Berrichon du Cher [46]. A total of 1099 male and female lambs born over 5 years (approximately 220 lambs per year) and allocated in 9 half-sib families (on average 122 lambs per family) were used in this study. The animals were reared and experiments were conducted at the INRA experimental farm of La Fage (Roquefort sur Soulzon, France). All the animals were born in the spring and reared exclusively outdoors with their dams under extensive conditions (approximately 1 ewe/ha). The farming system and management characteristics have previously been described by Gonzalez *et al*. [47]. All lambs were identified at birth using ear tags and weaned at 75 ± 4 days of age. After weaning, lambs were maintained as a single flock and had minimal contact with humans until the period of the behavioral tests.

### Ethics statement

The experiments described here and performed before 2013 fully comply with the legislation on research involving animal subjects according to the European Union Council Directive of November 24, 1986 (86/609/EEC). The investigators carrying out the experiments were certified by the relevant French governmental authority. All experimental procedures were approved by the Animal Care Committee of Aveyron Veterinary Center under guidelines for the care and use of experimental animals established by the French Ministry of Agriculture ethics policy (agreement n°12-004).

### General experimental design

Experimental lambs were individually exposed to a series of 4 reactivity tests combining social attraction or isolation, exposure to humans/handling, confinement and novelty: 1) an arena test, 2) a corridor test, 3) an isolation box test, and 4) a shearing test. The arena and corridor tests were adapted from the tests developed by Boissy and colleagues [25]. The isolation box test was developed by Murphy [48] and has been used for experimental selection for temperament in Merino sheep [30]. The shearing procedure was standardized to assess cortisol concentrations in response to an aversive handling event.

The arena and corridor tests were performed indoors between 10 and 20 days after weaning (about 30 animals per day for both tests). The day before being tested, the lambs to be tested were removed from the flock and penned under a shelter where they had permanent access to hay, concentrate and water (except during the actual testing time). At the end of the testing day, the lambs were returned to their flock. The isolation box test was carried out 3 weeks after weaning for all the animals of the year’s flock. One week later, the shearing test was carried out on the female lambs only (i.e. male lambs were fattened and sold without being shorn).

#### Arena test

The aim of the arena test (AT) was to evaluate the social motivation of the lambs and their reactivity to a motionless human. The test consisted of two successive phases evaluating 1) reactivity to social isolation and 2) reactivity to a conflict between social attraction and avoidance of a motionless human. The test pen consisted in an unfamiliar enclosure virtually divided into 7 zones (zone 7 being the zone nearest to conspecifics) as described in detail by Ligout *et al*. [28]. In our experiment, the first phase of the test (arena test phase 1, AT1) began once the tested animal joined its flock-mates located behind a grid at the opposite side of the arena (time duration for joining: lower than 15 sec). At this time, an opaque panel was pulled down (from the outside of the pen) between the flock-mates and the tested lamb to prevent visual contact. After one minute the phase 1 stopped and the panel was pulled up so the lamb could see its flock-mates again. Once the lamb had returned near to its flock-mates, or after 1 minute if the lamb did not do so, a non-familiar human slowly entered the arena through a door located near the pen of the flock-mates, and stood 20 cm in front of the grid separating the arena from the lamb’s flock-mates. The second phase (arena test phase 2, AT2) began once the human was in place and lasted for a further 1 minute.

#### Corridor test

The corridor test (CT) evaluated reactivity to social isolation and to an approaching human. It consisted of two successive phases: 1) reactivity to social isolation, and 2) reactivity to a walking human (i.e. repeated approaches). The test pen consisted in a closed, wide rectangular circuit and has been described in detail by Boissy *et al*. [25]. The first phase (corridor test phase 1, CT1) began when the lamb entered the testing pen and lasted for 30 seconds. After that time a non-familiar human entered the testing pen and the second phase (corridor test phase 2, CT2) started and lasted 1 minute. During this phase, the human walked at a regular speed through the corridor (the corridor was divided into 6 virtual zones and one zone was crossed every 5 seconds) until two complete tours had been achieved. Every five seconds throughout this phase, the zones in which the human and the animal were located were recorded, and the walking human recorded with a stopwatch the total duration during which the head of the lamb was visible.

#### Isolation box test

The aim of the isolation box test (IBT) was to evaluate the reactivity of the lambs to social isolation, novelty and confinement [30]. A 1.5 × 1.5 × 1.5 m wooden box resting on four tires was placed in the animals’ pasture. Lambs were then moved as a group into a waiting pen near the box and then each lamb was individually introduced into the isolation box and maintained within the box for 30 seconds. The agitation of the isolated lamb was recorded using an electronic ‘agitatometer’ (Physiology Electronics, University of Western Australia) registering each vibration of the box resulting from the movements of the lamb. In order to minimize any potential variation along the time, a calibration of the agitometer was performed before each testing day: the sensitivity of the agitometer was readjusted by using a calibration unit (University of Western Australia) that was placed on the floor of the IBT and produced repetitive and standardized vibrations simulating the action of a lamb whilst in the box.

#### Shearing test

The shearing test assessed the rise in cortisol levels due to the stress induced by the shearing procedure. The test was carried out only on females. The ewe-lamb flock was gathered in a waiting area. One investigator caught the lamb to be shorn 15 seconds before the shearer finished shearing the previous animal and maintained it motionless 2 meters away from the shearer until it was its turn. The shearer then caught the animal and the shearing procedure began and lasted no longer than 1.5 minutes. The lamb was then released in a corridor made of metal hurdles in contact with its flock mates. Fifteen minutes after being sheared, another investigator caught the animal and maintained it motionless with its head up while a third investigator took a blood sample from jugular vein. The lamb was then released to pasture. The blood sample was centrifuged within a few minutes and plasma samples were transferred and frozen to be later assayed for cortisol concentration (CORT) by radioimmunoassay [49].

#### Behavioral traits

Arena and corridor tests were video recorded. Locomotor activity, vigilance postures, and behaviors to the flock-mates or the human, were measured afterwards using the video software The Observer 6.0 (Noldus). Locomotor activity was assessed by measuring the number of zones crossed during arena test phases 1 and 2 (AT1/2-LOCOM) and corridor test phase 1 (CT1-LOCOM). The overall degree of agitation during the isolation box test was measured objectively using an electronic ‘agitation meter’ (IBT-LOCOM). Vigilance postures (i.e. animal motionless, head in an upright position and ears perpendicular to the head) were measured during arena test phase 1 (AT1-VIGIL). The proximity to flock-mates and the human during arena test phase 2 was calculated using the following formula: AT2-PROX = (time spent in zone 1 × 0/6) + (time spent in zone 2 × 1/6) + … + (time spent in zone 7 × 6/6). Deviation from normal distribution is minimized using this formula compared with the formula described by Ligout *et al.* [28]. The mean distance separating the human and the lamb and the time during which the human saw the lamb were measured for corridor test phase 2 (CT2-DIST and CT2-SEEN, respectively). During the actual tests, an investigator counted the lamb’s vocalizations from outside the pen using a laptop: number of times the animal bleated with an open mouth (high bleats, AT1/2-HBLEAT, CT1-HBLEAT, IBT-HBLEAT), and number of times the animal bleated with a closed mouth (low bleats, AT1/2-LBLEAT, CT1-LBLEAT). Only high bleats were recorded during arena test phase 2 and the isolation box test.

#### Statistical handling

Deviations from normality of row data were tested using the Kolmogorov–Smirnov test (Univariate procedure of SAS, SAS Institute Inc.). Several raw measures were transformed in order to minimize major deviations from the normal distribution. Square root transformation was applied to AT1/2-HBLEAT, CT1-HBLEAT, IBT-HBLEAT and IBT-LOCOM. Logarithmic transformation was applied to AT1/2-LBLEAT, CT1-LBLEAT and CORT. A multivariate analysis was performed to take into account the multidimensional aspect of behavioral responses. Principal component analysis (PCA), followed by orthogonal rotation was carried out using SAS^®^ software (FACTOR procedure, version 8.1, SAS^®^ Institute Inc., Cary, NC, USA). Factor scores were then calculated for each lamb and used in subsequent analyses. In addition, we constructed four synthetic variables using PCA (PRINCOMP procedure in the SAS^®^ software). Each PCA was performed for a set of similar behavioral variables across the three behavioral tests. The first component of each PCA, explaining the largest part of total variance, was defined as a synthetic variable. Three synthetic variables were specific to the reactivity to social isolation: high bleats (ISO_HBLEAT), locomotion (ISO_LOCOM) and low bleats (ISO_LBLEAT). One synthetic variable was specific to the reactivity to a human: the tolerance to being approached when the lamb was free to flee (HUMAPPRO). The correlations between original measurements and the 4 synthetic variables ranged between 0.64 and 0.93. The synthetic variables accounted for 46% to 87% of the total variability. Analyses of variance using the GLM procedure of the SAS^®^ software were performed on the original and synthetic variables to assess differences between the lambs for different rearing year, sex, litter size born and litter size reared, and the age of the mother. Phenotypes were then corrected for identified fixed effects prior to subsequent genetic analyses (Additional file 1). Heritability estimates were generated for each trait by restricted maximum likelihood (REML) methodology using univariate analyses with VCE 4.2.5 software (Neumaier and Groeneveld, 1998). Random effects in the mixed models included a direct genetic effect (animal), a maternal permanent environment effect and a litter permanent environment effect.

### SNP genotypes

1038 lambs (out of the 1099 lambs phenotyped) were genotyped as well as their nine respective sires using the Illumina OvineSNP50 BeadChip (comprising 54,241 SNPs). Individuals with a call rate (i.e. number of called SNPs per sample over the total number of SNPs in the data set) below 98% and with Mendelian inconsistencies (i.e. no alleles shared between it’s a sire and its progeny for a given SNP) were discarded (104 and 9 lambs, respectively). SNP quality was also checked as described by Sallé *et al*. [44]. 5448 SNPs with a call rate lower than 97%, a minor allele frequency below 1% or inconsistent Hardy-Weinberg disequilibrium were discarded. Furthermore, 3095 SNPs were discarded due to a too high recombination rate or because more than 50% of heterozygous sire’s offspring were heterozygous. Sex chromosomes were not included in the analysis. Finally, 934 individuals and 40,725 autosomous SNPs were retained for QTL analyses.

### Methods for QTL detection

The QTLmap software was used to search for QTLs using linkage, association and joint linkage and association analyses [50].

#### Linkage analysis

Data were analyzed using linkage analyses (LA) by interval mapping within each sire family. The presence of a QTL was tested against the null hypothesis (absence of a QTL) at every 0.1-cM interval by likelihood computation. Chromosome-wise significance levels were computed for each chromosome and trait by testing with 3000 to 10,000 permutations [51]. Genome-wise thresholds were obtained using the Bonferroni correction (1-P_genome wise_) = (1-P_chromosome wise_)^n^ where n is the number of chromosomes (i.e. 26 autosomous chromosomes in sheep) [52]. Confidence intervals were determined using the “2 lod drop off” criterion and assuming 1 LOD = 4.61 LRT [53].

#### Association analysis and joint linkage and association analysis

Genome-wide association (GWAS) analysis was performed for the whole population genotyped using the existing linkage disequilibrium (LD) [54]. The LD linear model (named LD decay model) developed by Legarra et al. [55] was fitted to our data. The advantage of this LD method is that it be used with estimated haplotype effects.

Joint analysis (LDLA), considering simultaneously linkage association and linkage disequilibrium, was performed to take advantage of both pedigree and LD [55]. The LDLA model considered the sire haplotype effects of the LD model in addition to sire QTL effects.

Finer mapping was achieved with both GWAS and LDLA approaches than with linkage analysis alone. Assuming approximately 10 SNPs per cM in our study, the limit of accuracy may therefore be the LD pattern in our data. For both GWAS and LDLA analyses, a haplotype size of 4 SNPs was used. When haplotype frequency was lower than 1%, haplotypes were considered to belong to a rare haplotype group. The chromosome-wise p-values were estimated assuming that, conditional on the QTL position, the likelihood ratio test statistics followed a χ2-distribution with k degrees of freedom, k being the number of genetic effects [56]. In our study, k was equal to the number of haplotypes for GWAS and the number of haplotypes plus the number of families for LDLA. Genome-wise p-values were obtained using the Bonferroni correction assuming 26 chromosomes were analyzed (i.e. 26 independent tests) [52, 57].

## Results

### Phenotypes

Descriptive statistics of behavioral traits are summarized in Table 1. Locomotion in arena test phase 1 and agitation in the isolation box test were approximately 3 to 4 fold higher than in arena test phase 2 and corridor test phase 1. The highest and lowest numbers of high bleats were recorded in arena test phase 1 and 2, respectively, while the number of high bleats recorded in corridor test phase 1 and the isolation box test were intermediate. The number of low bleats was low whatever the test (in average less than 2.6 low bleats). Lambs spent in average one third of test duration in vigilance postures during arena test phase 1. Proximity to flock-mates and the human in arena test phase 2 was in average 27 s, which is approximately 2.2 fold lower than the maximum proximity duration (60 s). The mean distance separating the human and the lamb in corridor test phase 2 was 5.4 m and the mean time during which the human could see the lamb was 11 s. The mean cortisol concentration in response to the shearing procedure was 46.7 ng/ml. Factor analysis of the data revealed that four main factors accounted for 57.1% of the variability. Orthogonal rotation resulted in the factor loadings shown in (Additional file 2: Table S4). Factor 1 had high positive loadings for the frequency of the lamb to perform high bleats whatever the test and was designated “High Bleats”. Factor 2 had high positive loadings for proximity to a human and time during which the human could see the lamb and a negative loading for the mean distance separating the human and the lamb. Factor 2 was designated “Reactivity to humans”. Factor 3 had high positive loadings for the number of zones crossed whatever the test and a negative loading for vigilance postures. Factor 3 was designated “Locomotor activity”. Factor 4 had high positive loadings for the frequency of the lamb to perform low bleats whatever the test and was designated “Low Bleats”.

**Table 1:** **Summary statistics for the original and transformed measures recorded in lambs individually exposed to the behavioral tests**

Variable	n	Description	Original data			Transformed data			
			Mean (± SD)	Min	Max	Mean (± SD)	Min	Max	Transformation
AT1-LOCOM	1066	number	19.83 (8.80)	1	50				none
AT2-LOCOM	1066	number	4.84 (4.15)	1	36				none
AT1-HBLEAT	1099	number	10.59 (7.06)	0	40	2.95 (1.38)	0	6.33	Square root
AT2-HBLEAT	1099	number	2.50 (3.53)	0	26	1.09 (1.15)	0	5.10	Square root
AT1-LBLEAT	1099	number	2.65 (3.21)	0	21	0.41 (0.36)	0	1.34	Log
AT2-LBLEAT	1099	number	1.09 (2.02)	0	12	0.20 (0.29)	0	1.11	Log
AT1-VIGIL	1066	duration (s)	20.34 (9.82)	0	51.76				none
AT2-PROX	1033	duration (s)	26.7 (16.94)	0	60				none
CT1-LOCOM	1099	number	5.92 (2.21)	1	14				none
CT1-HBLEAT	1099	number	3.20 (3.39)	0	22	1.38 (1.14)	0	4.69	Square root
CT1-LBLEAT	1099	number	1.70 (1.99)	0	10	0.33 (0.30)	0	1.04	Log
CT2-DIST	1099	distance (m)	5.39 (1.16)	1.75	9				none
CT2-SEEN	1099	duration (s)	10.55 (7.18)	0	59				none
IBT-LOCOM	1094	number	17.81 (15.50)	0	149	3.86 (1.70)	0	12.21	Square root
IBT-HBLEAT	1094	number	5.62 (5.03)	0	35	2.01 (1.26)	0	5.92	Square root
CORT	477	concentration (ng/ml)	46.74 (18.48)	16	127.90	1.64 (0.17)	1.20	2.11	Log

### Linkage analysis

Eleven significant QTLs reaching the 1% chromosome-wise (CW) threshold and 10 significant QTLs reaching genome-wise (GW) thresholds (i.e. 5%, 1% or 0.1%) were mapped on OAR 5, 6, 10, 12, 13, 16, 17, 20, 21 and 24 (Table 2). Seventeen of these 21 QTLs were related to vocalizations (AT2/CT1/IBT/ISO-HBLEAT, FACTOR1, AT1/CT1/ISO-LBLEAT, FACTOR4), two QTLs were related to locomotor activity (AT2-LOCOM), one was related to the mean distance separating the human and the lamb (CT2-DIST) and one to the reactivity to humans (FACTOR2). In addition, 28 significant QTLs reaching the 5% CW threshold were detected and mapped on 14 chromosomes (Additional file 3: Table S1). Most of these 28 QTLs were related to vocalizations (14 out of the 28 significant QTLs, Additional file 3: Table S1); the other QTLs were related to locomotor activity, proximity to flock-mates and the human, the mean distance separating the human and the lamb and vigilance postures. A significant QTL reaching the 5% CW threshold associated with cortisol concentrations (CORT) was mapped on OAR19. Mean QTL effects ranged from 0.18 to 0.30 phenotypic standard deviations.

**Table 2:** **Summary of QTLs detected in Linkage Analyses studies**

OAR	Trait	Signifi-cance ^1^	Level	Position ^2^(Mb)	Confidence Interval	Average QTL Effect ^3^
5	FACTOR1	**	CW	93.8	92.0 - 95.3	0.26
5	ISO_HBLEAT	**	CW	96.8	92.8 – 97.3	0.22
5	CT1-HBLEAT	*	GW	93.7	92.0 – 95.4	0.25
5	IBT-HBLEAT	*	GW	52.6	47.0 – 55.0	0.22
6	FACTOR2	**	CW	111.7	111.6 - 111.8	0.22
10	AT2-LOCOM	**	CW	16.1	14.2 – 17.8	0.20
12	FACTOR4	*	CW	69.0	63.7 - 71.1	0.20
12	ISO_LBLEAT	*	GW	67.8	65.6 – 70.9	0.22
12	AT1-LBLEAT	*	CW	70.4	66.4 – 71.2	0.27
12	CT1-LBLEAT	**	CW	27.5 (68.4) (68.4)	26.1 – 29.1	0.21
12	CT2-DIST	*	CW	33.1	29.7 – 39.4	0.23
13	FACTOR1	*	CW	41.4	37.1 - 43.1	0.16
13	ISO_HBLEAT	*	CW	39.7	37.5 – 43.9	0.22
13	CT1-HBLEAT	**	GW	41.4	40.5 – 44.0	0.27
16	FACTOR1	***	GW	45.2	41.9 - 48.1	0.24
16	ISO_HBLEAT	**	CW	43.7	41.3 – 49.8	0.24
16	AT1-HBLEAT	*	CW	47.7	39.5 – 49.8	0.21
16	AT2-HBLEAT	***	GW	45.1	41.7 – 46.6	0.30
16	IBT-HBLEAT	*	CW	45.8	42.2 – 48.8	0.25
16	CT2-DIST	**	CW	34.4 (48.0)	33.0 – 36.8	0.24
17	CT1-HBLEAT	**	CW	39.0	35.1 – 42.3	0.19
17	ISO_LBLEAT	*	GW	33.3	30.5 – 36.8	0.26
17	IBT-LOCOM	*	CW	66.4	64.7 – 72.0	0.21
19	CORT	*	CW	41.0	38.9 – 43.2	0.27
20	IBT-LOCOM	**	CW	38.3	36.9 – 43.2	0.26
21	FACTOR4	***	GW	47.6	45.9 - 48.5	0.27
21	AT2-HBLEAT	*	CW	36.0	14.7 – 37.1	0.19
21	ISO_LBLEAT	**	GW	39.3	38.1 – 40.0	0.26
21	AT1-LBLEAT	**	CW	39.3	37.9 – 41.6	0.22
21	CT1-LBLEAT	*	GW	47.3	46.5 – 48.4	0.26
24	CT1-LBLEAT	**	CW	9.8	9.1 – 11.7	0.19
26	FACTOR2	*	CW	42.45	41.9 - 44.7	0.21

Only the significant QTLs reaching the 1% chromosome-wise threshold or the genome-wise thresholds are listed in the table. QTLs reaching the 5% chromosome-wise threshold are also reported if the QTL was also detected for the same trait by association analysis (GWAS). ^1^: *, p < 5%; **, p < 1%; ***, p < 0.1%. ^2^: the position of a second significant QTL is indicated between parentheses. ^3^: average QTL effect given in phenotypic standard deviation. CW, Chromosome Wide; GW, Genome Wide.

**Figure 1:**
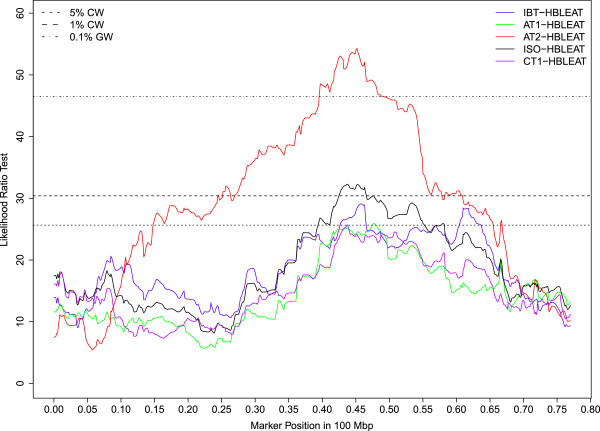
**Likelihood profiles of the linkage analyses on OAR16 for high bleat vocalization in the isolation box test (IBT-HBLEAT), arena test phase 1 (AT1-HBLEAT), arena test phase 2 (AT2-HBLEAT), corridor test phase 1 (CT1-HBLEAT) and for the synthetic variable ISO-HBLEAT.** Horizontal lines indicate average thresholds for the five HBLEAT traits at the 5% chromosome wide threshold (5% CW), 1% chromosome wide threshold (1% CW) and 0.1% genome wide threshold (0.1% GW).

**Figure 2:**
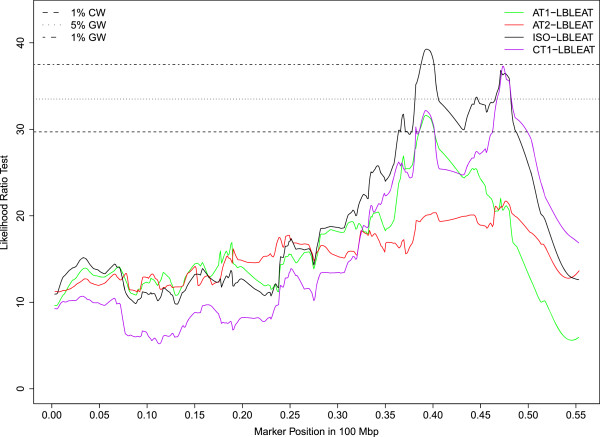
**Likelihood profiles of the linkage analyses on OAR21 for low bleat vocalization in the arena test phase 1 (AT1-LBLEAT), arena test phase 2 (AT2-LBLEAT), corridor test phase 1 (CT1-LBLEAT) and for the synthetic variable ISO-LBLEAT.** Horizontal lines indicate average thresholds for the four LBLEAT traits at the 1% chromosome wide threshold (1% CW), 5% genome wide threshold (5% GW) and 1% genome wide threshold (1% GW).

**Figure 3:**
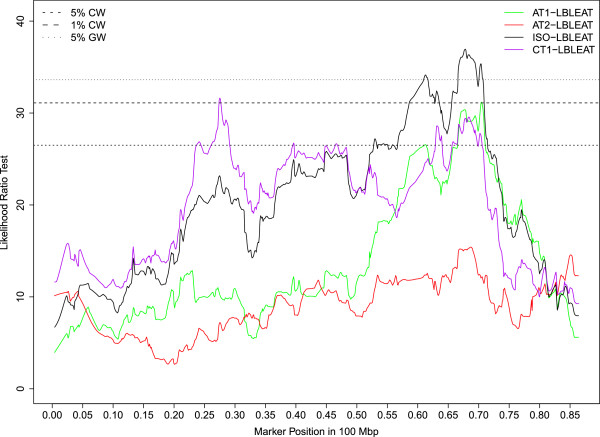
**Likelihood profiles of the linkage analyses on OAR12 for low bleat vocalization in the arena test phase 1 (AT1-LBLEAT), arena test phase 2 (AT2-LBLEAT), corridor test phase 1 (CT1-LBLEAT) and for the synthetic variable ISO-LBLEAT.** Horizontal lines indicate average thresholds for the four LBLEAT traits at the 5% chromosome wide threshold (5% CW), 1% chromosome wide threshold (1% CW) and 5% genome wide threshold (5% GW).

Seventy percent of the QTLs mapped to 6 chromosomes (OAR5, 12, 13, 16, 17, 21) and almost all of the significant QTLs reaching either the 1% CW threshold or GW thresholds were clustered on 4 of these 6 chromosomes (OAR5, 12, 16, 21). On OAR16, a QTL region mapped between 39.5 and 49.8 Mb was repeatedly associated with high bleat vocalizations (FACTOR1) expressed in sheep subject to social isolation (ISO_HBLEAT, AT1-HBLEAT, IBT-HBLEAT) or in the presence of a human (AT2-HBLEAT) (Figure 1). A QTL for the mean distance separating the human and the lamb (CT2-DIST) also mapped on OAR16 in the confidence interval found for high bleat vocalizations. Four QTLs related to high bleat vocalizations (IBT/CT1/ISO-HBLEAT, FACTOR1) were also mapped on OAR5 but in two distinct regions with no overlap of the confidence intervals. A QTL region on OAR21 (10 Mb large) was significantly associated with low bleat vocalization traits (FACTOR4, ISO_LBLEAT, AT1-LBLEAT, CT1-LBLEAT) (Figure 2). Two other QTLs reaching the 5% CW threshold and related to high bleat vocalization (AT2-HBLEAT, CT1-HBLEAT) were also mapped to a location 2 Mb from the previous OAR21 region (Additional file 3: Table S1). On OAR12, a QTL region with a narrow confidence interval was associated with low bleat traits (FACTOR4, ISO_LBLEAT, AT1-LBLEAT, CT1-LBLEAT) (Figure 3).

### Association analysis (GWAS) and joint linkage and association analysis (LDLA)

The QTLs reaching the 1% CW significance threshold or the GW significance thresholds (5%, 1% or 0.1%) found using association analysis (GWAS) are reported in Table 3. QTLs reaching the 5% CW threshold are also reported in Table 3 if the QTL was also found for the same trait using linkage analysis. Complete lists of QTLs found by GWAS or joint linkage and association analysis (LDLA) are reported in Additional file 4: Table S2 and Additional file 5: Table S3, respectively.

**Table 3:** **Summary of QTL detected in GWAS**

OAR	Trait	Signifi-cance ^1^	level	Pos ^2^(Mb)	Confidence interval	No. haplo-types	Flanking markers ^3^	
2	FACTOR1	*	GW	143.8	143.7 - 143.9	11	s01640.1	OAR2_143893183.1
2	ISO_HBLEAT	**	CW	143.8	143.7 - 151.1	11	s01640.1	OAR2_143893183.1
3	FACTOR4	**	CW	136.8	136.7 - 136.9	9	OAR3_136802294.1	OAR3_136865536.1
5	FACTOR1	*	CW	92.1	92.0 - 92.2	11	OAR5_92064206.1	OAR5_92169254.1
5	AT1-LBLEAT	**	CW	80.3	74.3 - 80.4	10	OAR5_80246247.1	OAR5_80304302.1
6	FACTOR2	**	CW	110.0	100.1 - 113.2	9	s29906.1	OAR6_110056838.1
7	ISO_LBLEAT	**	CW	52.1	43.5 - 52.2	9	OAR7_52124140.1	OAR7_52157129.1
8	AT1-LBLEAT	*	GW	69.1	60.9 - 69.2	10	OAR8_69105959.1	OAR8_69151787.1
12	FACTOR4	**	GW	56.3	56.2 - 56.4	13	s63508.1	OAR12_56302501_X.1
12	AT1-LBLEAT	*	CW	56.3	53.2 - 80.5	13	s63508.1	OAR12_56302501_X.1
12	AT2-LBLEAT	*	GW	56.3	56.1 - 61.6	13	s63508.1	OAR12_56302501_X.1
12	CT2-DIST	**	CW	32.6	25.7 - 32.7	10	OAR12_32559849.1	OAR12_32617508.1
12	ISO_LBLEAT	**	GW	56.2	56.1 - 56.3	13	s63508.1	OAR12_56302501_X.1
13	FACTOR1	**	CW	45.6	39.3 - 51.2	13	s73104.1	s43103.1
13	FACTOR4	**	CW	64.3	62.5 - 64.4	9	s00952.1	s70439.1
13	CT1-LBLEAT	*	CW	20.5	20.4 - 29.3	12	s30126.1	OAR13_20537279.1
13	ISO_HBLEAT	*	CW	45.6	34.0 - 45.7	13	s73104.1	s43103.1
15	AT1-LBLEAT	***	CW	74.8	74.7 - 74.9	10	OAR15_74759937.1	OAR15_74809345.1
16	FACTOR1	***	GW	43.9	43.8 - 46.3	11	OAR16_43833978.1	OAR16_43916302.1
16	AT1-HBLEAT	*	CW	46.3	42.7 - 46.4	13	OAR16_46290531.1	OAR16_46325523.1
16	AT2-HBLEAT	**	GW	44.3 (20.1)	44.0 - 45.7	14	OAR16_44325630.1	s23014.1
16	CT2-DIST	*	CW	45.4	45.3 - 46.3	12	OAR16_45398511.1	s17055.1
16	IBT-HBLEAT	*	CW	46.3	44.4 - 46.8	13	OAR16_46290531.1	OAR16_46325523.1
16	ISO_HBLEAT	*	GW	43.9	43.5 - 46.7	11	OAR16_43833978.1	OAR16_43916302.1
17	FACTOR2	***	CW	5.9	5.8 - 7.3	11	OAR17_5938733.1	OAR17_5979442.1
17	IBT-LOCOM	*	CW	40.6	22.7 - 40.7	9	OAR17_40579701.1	OAR17_40700344.1
17	ISO_HBLEAT	*	CW	12.7	12.6 - 15.0	12	s42157.1	OAR17_12809597.1
19	AT2-LOCOM	***	GW	18.9	18.8 - 19.0	9	OAR19_18888791.1	s24963.1
19	CORT	*	CW	39.1	39.0 - 41.8	7	OAR19_39084409.1	OAR19_39123291.1
20	CT2-SEEN	**	CW	7.6	7.5 - 7.7	10	OAR20_7626319.1	OAR20_7794638.1
21	FACTOR1	**	CW	38.0	36.6 - 40.4	9	OAR21_38037300_X.1	OAR21_38087037.1
21	AT1-LBLEAT	*	GW	7.6	7.2 - 7.7	14	s54902.1	OAR21_7730122.1
21	AT2-HBLEAT	*	CW	38.0	25.3 - 45.1	9	OAR21_38037300_X.1	OAR21_38087037.1
21	ISO_HBLEAT	*	CW	43.1	36.6 - 45.1	10	OAR21_43118557.1	s61819.1
23	FACTOR2	*	GW	32.2	32.1 - 32.4	12	OAR23_32137172.1	s55273.1
23	AT2-LOCOM	*	GW	54.1	51.6 - 54.2	10	OAR23_54064411.1	s31567.1
23	CT2-SEEN	**	CW	32.3	27.9 - 32.4	10	s55273.1	s74857.1
23	HUMAPPRO	**	CW	32.2	28.9 - 32.3	12	OAR23_32137172.1	s55273.1
26	FACTOR1	**	CW	6.4	4.5 - 9.5	10	s58657.1	DU481531_204.1
26	FACTOR2	*	CW	41.2	39.8 - 47.6	10	OAR26_41166096.1	s07654.1
26	ISO_HBLEAT	**	CW	45.4	45.3 - 46.8	13	s31164.1	s16475.1

Only the significant QTLs reaching the 1% chromosome-wise threshold or the genome-wise threshold are listed in the table. QTLs reaching the 5% chromosome-wise threshold are also reported if the QTL was also detected for the same trait by linkage analysis. ^1^: *, p < 5%; **, p < 1%; ***, p < 0.1%. ^2^: the position of a second significant QTL is indicated between parentheses. ^3^: SNPs flanking the haplotype with significant association. CW, Chromosome Wide; GW, Genome Wide.

Using GWAS, 16 haplotype-trait associations reached the 1% CW significance threshold and 12 haplotype-trait associations reached the GW significance thresholds (Table 3). Nineteen of these 28 QTLs were related to vocalizations (AT2/ISO-HBLEAT, FACTOR1, AT1/AT2/ISO-LBLEAT, FACTOR4). Seven QTLs were related to the mean distance separating the human and the lamb (CT2-DIST), or to the time during which the human could see the lamb (CT2-SEEN), or to a combination of both traits (HUMAPPRO, FACTOR2). Two QTLs were related to locomotor activity (AT2-LOCOM).

All the significant QTLs that reached the 1% CW threshold or GW thresholds found by GWAS were also detected by LDLA analysis (Additional file 5: Table S3). Among the 28 QTLs detected by GWAS, 9 were also found by linkage analysis. In addition, 72 significant QTLs reaching the 5% CW threshold were found by association analysis. Most of these QTL were related to vocalizations (43 of the 72 QTLs, Additional file 4: Table S2). All of the QTLs reaching the 5% CW significance threshold detected by GWAS that were found for the same trait by linkage analysis were also detected by LDLA (with a 1% genome-wise significance p-value for LDLA).

**Figure 4:**
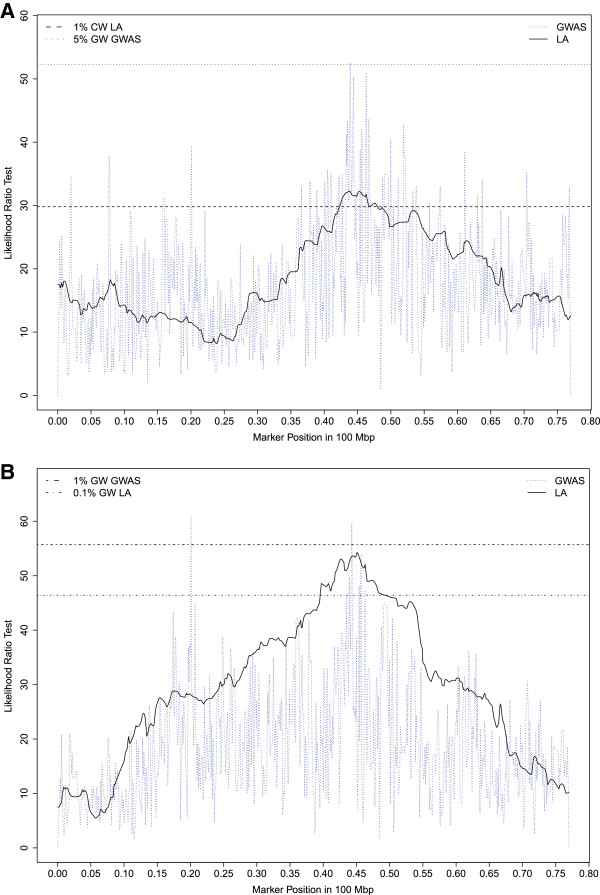
**Likelihood ratio test profiles on OAR16 for ISO-HBLEAT with the linkage or association analyses, Likelihood ratio test profiles on OAR16 for AT2-HBLEAT with the linkage or association analyses. A**: Horizontal lines indicate the 1% chromosome wide threshold for linkage analysis (1% CW LA) and the 5% genome wide threshold for association analysis (5% GW GWAS) for ISO-HBLEAT. **B**: Horizontal lines indicate the 0.1% genome wide threshold for linkage analysis (0.1% GW LA) and the 1% genome wide threshold for association analysis (1% GW GWAS) for AT2-HBLEAT.

**Figure 5:**
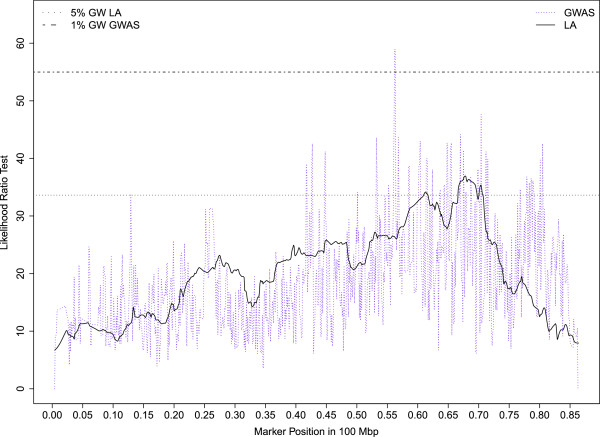
**Likelihood ratio test profiles on OAR12 for ISO-LBLEAT with the linkage or association analyses.** Horizontal lines indicate the 5% genome wide threshold for linkage analysis (5% GW LA) and the 1% genome wide threshold for association analysis (1% GW GWAS).

**Figure 6:**
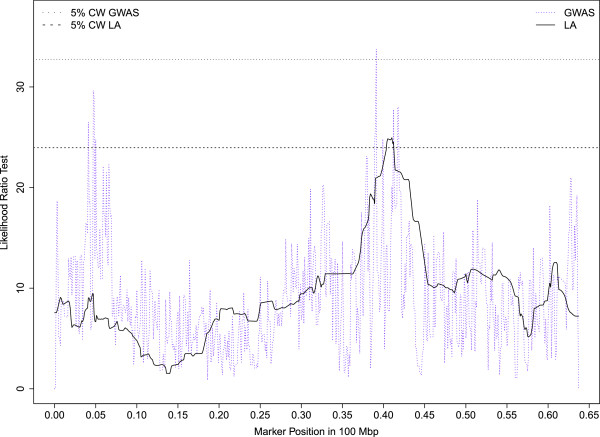
**Likelihood ratio test profiles on OAR19 for CORT with the linkage or association analyses.** Horizontal lines indicate the 5% chromosome wide threshold for linkage analysis (5% CW LA) and the 5% chromosome wide threshold for association analysis (5% CW GWAS).

Chromosomes OAR2, 12, 13, 16, 17 and 21 showed consistent associations using the three different analysis methods (i.e. 17 QTLs were detected whatever the analysis method). Three of these six chromosomes were already highlighted by LA (OAR12, 16, 21). Five associations related to high bleats mapped to the same QTL region on OAR16 as found with LA (AT1/AT2/IBT/ISO-HBLEAT, FACTOR1; 42.7 – 46.8 Mb). The likelihood ratio test profiles for ISO-HBLEAT and AT2-HBLEAT obtained on OAR16 with the linkage and association analyses are shown in Figure 4A and B, respectively. A QTL for the mean distance separating the human and the lamb (CT2-DIST) was also mapped on OAR16 within the confidence interval of the high–bleat region. Four associations related to low-bleating behavior (AT1/AT2/ISO-LBLEAT, FACTOR4) were found on OAR12, close to the confidence interval determined by LA. The likelihood ratio test profiles for ISO-LBLEAT obtained with linkage and association analyses on OAR12 are shown in Figure 5. Another QTL for the mean distance separating the human and the lamb (CT2-DIST) was also mapped on OAR12 within the region highlighted by LA for this trait. Three associations for vocalization behaviors (AT2/ISO-HBLEAT, FACTOR1) were found on OAR21 in the same QTL region as found with LA. Last, an association related to corticosterone concentrations was mapped on OAR19 in the same QTL region as found with LA for this trait (Figure 6).

**Figure 7:**
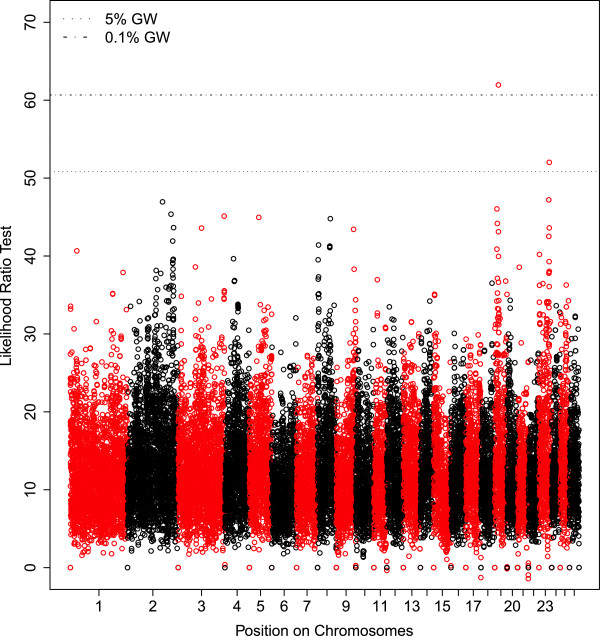
**Manhattan plot of the likelihood ratio test values obtained for AT2-LOCOM by GWAS.** The likelihood ratio test is plotted against SNP haplotype positions (four consecutive SNPs) along the genome (from chromosome one to 26). Horizontal lines indicate the 5% genome wide threshold (5% GW) or the 0.1% genome wide threshold (0.1% GW) for association analysis.

In addition, other chromosomes (OAR5, 7, 8, 15, 19, 20, 23 and 26) exhibited significant associations only found with both association analyses. Among these associations, it can be noted that locomotor activity (AT2-LOCOM, Figure 7) mapped to OAR19 and OAR23, the time during which the human saw the lamb (CT2-SEEN) mapped to OAR20 and OAR23, and the combination of the time during which the human saw the lamb and the mean distance separating the human and the lamb (HUMAPPRO, FACTOR2) mapped to OAR23.

## Discussion

Considering the existing genetic variability for behavioral traits previously reported in sheep [25, 35, 58], the aim of the present study was to provide the first characterization of the genetic architecture controlling behavioral reactivity to conspecifics and a human in domestic sheep. This was achieved not only by using dense molecular information (Ovine 50kSNP BeadChip) but also by studying a broad range of behavioral phenotypes and using complementary methods of analysis to detect robust and fine-mapped QTLs.

### The first QTLs for behavioral responses in domestic sheep

As far as we know, we have mapped the first published QTLs for behavioral traits in domestic sheep. Indeed, although the increased awareness to welfare standards in farm animals has led to an growing number of behavioral studies in livestock species [59], especially in sheep, cattle, pigs, poultry and fish [26, 60–62], investigations on the genetics of behavior in livestock species are scarce. QTLs for behavior have already been reported in cattle [40, 41], pigs [63], poultry [64] and fish [38] but not so far in sheep, albeit for a recent QTL analysis of personality in wild bighorn sheep in which Poissant *et al*. reported two suggestive QTLs for docility and boldness in response to capture on chromosomes 2 and 6, respectively [65]. However, it is difficult to compare our findings with the behavioral QTLs in bighorn sheep because of the long and divergent evolution history between the *Ovis aries* (domestic sheep) and *Ovis canadensis* (bighorn sheep) species in the *caprinae* subfamily [66].

In our study, we were particularly interested in detecting QTLs for behavioral reactivity and cortisol response in sheep exposed to social isolation, with or without human presence. Both linkage and LD-based analyses resulted in mapping many QTLs involved in reactivity to social and/or human challenges. Findings with the various methods were consistent only for a few regions with several correlated traits mapping to a limited region (OAR12, 16, 21, 23) and/or a high level of significance (OAR5, 8, 12, 13, 16, 19, 21). The QTL regions detected on OAR12, 16 and 21 were both associated with several correlated traits and showed high levels of significance.

These QTL regions on OAR12, 16 and 21 appeared as the most interesting QTL regions for social reactivity and were mostly associated with low and high bleats, those traits were found the most heritable (Additional file 6: Table S5). Interestingly, QTLs for vocalization have also been reported in cattle [41]. Gutiérrez-Gil *et al*. found that traits related to the frequency of vocalizations in a social separation test were among the traits with the highest number of QTLs. Other QTLs for behavioral responses to social challenges have been identified in livestock productions (for review see [37]) but were associated with the time spent in a social zone (sociability test), interaction with their mirror image (mirror test) and attack latency (resident intruder test) in chicken [39] or shoaling tendency in zebra fish [38].

Considering original behavioral variables, only the QTLs mapped for similar behavioral traits assessed in the different behavioral tests (i.e. arena, corridor and isolation box tests) were found to overlap. This is particularly the case for the QTL region on OAR16 which is associated with high vocalization measurements, whatever the behavioral test. Similarly, the QTL regions on OAR12 and 21 were both associated with each of the low vocalization measurements, whatever the behavioral test. These results are consistent with the high genetic correlations found between high vocalization measurements and low vocalization measurements. However, the QTLs for high vocalization measurements and low vocalization measurements did not overlap, consistently with the low genetic correlations found between low- and high- bleating behaviors (-0.3 ± 0.06) . Such overlapping of the QTL regions associated with similar traits assessed in the different tests may be due to the stimulus involved in the tests. Indeed, the behavioral responses in the different tests could be triggered by the common stimulus that is social isolation in a novel environment, either with or without human proximity.

Consistently with the results obtained for original behavioral variables, no overlaps were detected for the QTLs associated with the 3 specific synthetic variables for reactivity to social isolation (ISO-HBLEAT/LBLEAT/LOCOM) or with FACTORS (FACTOR1, 3 and 4). Again, this is consistent with the low genetic correlations found between vocalizations and locomotion (0.2 ± 0.06). All these results suggest that the reactivity to social isolation measured through high and low vocalizations and locomotion is influenced by different loci. This is in line with a previous study in cattle that demonstrated that temperament-related traits measured in a flight-from-feeder test and a social separation test have different underlying genetic causes [41]. Gutiérrez-Gil et al. reported no overlapping QTLs for flight distance, walking-escaping-running, standing in alert, and vocalizations. The results of anxiety-related behavior studies in mice also suggest that different genetic factors may regulate different aspects of behavior [67]. Using a set of behavioral tests of anxiety including the open-field, elevated plus-maze, square maze, light–dark box and mirror chamber, Turri *et al.* [67] reported QTLs that influenced behavior in all tests: a first QTL for the general level of motor activity, a second QTL for avoidance behavior and a third QTL for exploratory behavior.

Changes in the social environment can also involve the presence of a human. In our study, we measured reactivity to human presence during a second phase in both arena and corridor tests (traits AT2 and CT2, respectively). Although no QTLs could be detected for locomotion in response to social isolation (phase 1 of arena and corridor tests), we mapped QTLs for locomotion in the presence of a human (AT2-LOCOM) to OAR10, 19, and 23. On OAR19, we determined a haplotype with a large effect (2 phenotypic standard deviations) on locomotion in the presence of a human; however the frequency of this haplotype was low in our study (1%). In addition, we mapped QTLs for flight distance from a walking human (CT2-DIST), as measured in the corridor test, to chromosomes 12 and 16. The QTL for this trait that was mapped on OAR12 did not overlap with the QTL associated with low bleats. However, on OAR16, the QTL for CT2-DIST overlapped with the QTL associated with high bleats. Nevertheless, the effects of this QTL on OAR16 may not be pleiotropic for both behaviors because the QTL segregated in different set of families depending on the trait. These QTLs found here to be associated with reactivity to humans in sheep were consistent with those detected for similar traits in cattle. Indeed, co-locating QTLs are involved in the determinism of a mobility score and habituation to handling [40], and QTLs that influence the unprovoked flight from a feeder [41] have been found in dairy cattle and/or beef cattle.

We mapped QTLs on OAR16 and OAR21 for high vocalizations and on OAR12 for low vocalizations both recorded in the arena test phase 2. On chromosome 16, the QTL for high vocalizations in the second phase of the arena test overlapped with both of the QTLs for high vocalizations in the first phase of the arena test and the box test. Similarly on chromosome 12, the QTLs for low vocalizations in phases 1 and 2 of the arena test overlapped. These results suggest that the QTLs on chromosomes OAR12, 16 and 21 associated with vocalizations may reflect the reaction to social isolation rather than human presence, whatever the test. In addition, QTLs for the synthetic variable related to the reaction to an approaching human (HUMAPPRO) and for FACTOR2 did not overlap with QTLs for the 3 specific synthetic variables for reactivity to social isolation and for FACTOR1, 3 and 4. These results suggest that reactivity to humans and reactivity to social isolation are influenced by different loci. This is consistent with the low genetic correlations between vocalization behaviors and reactivity to humans (0.38 ± 0.1 and -0.36 ± 0.04 for high and low vocalizations, respectively) but somewhat contradictory with the high genetic correlations found between locomotion and reactivity to humans (-0.57 ± 0.04).

### Behavioral reactivity in sheep under polygenic influence

Our results using ovine SNP data support the hypothesis that behavioral traits in domestic sheep are under polygenic influences similarly to other complex traits [68, 69] without there being a major effect locus. Nevertheless, several of the QTLs mapped in the present study could probably act together to account for the substantial genetic variation observed for behavioral traits (Additional file 6: Table S5). For the 16 traits considered in the present study, linkage analysis mapped 49 QTLs on 17 chromosomes and only 10 QTLs reached the genome-wise significance threshold. Similarly, 100 QTLs were detected on 24 chromosomes with LD-based analysis, with only 12 QTLs reaching the genome-wise significance threshold. The present results from linkage analysis and LD-based analysis are in agreement with other QTL mapping studies for behavioral traits in which relatively few significant QTLs [41] or only suggestive QTLs were detected [65]. Ten years ago, Jonathan Flint [19] reviewed the QTLs that influence behavior in rodents and concluded that a great number of loci had been detected.

The QTL effects detected in the present study explain less than 10.6% of the phenotypic variation and are consistent with previous QTL studies of behavioral traits in livestock species and rodents. Indeed, Canario *et al*. [37] recently reviewed the knowledge obtained so far on the quantitative genetics of behavioral traits in four livestock species: cattle, pigs, poultry and fish. Whatever the species and behavioral traits, phenotypic variance explained by QTLs was always lower than 10%. For instance, the percentage of the variance explained by the QTLs identified to affect temperament traits in cattle ranged from 3.7% to 8.41% [41]. In poultry, several QTLs were found for locomotion, exploration and vocalization as measured by the open field test and for distance covered to join cage-mates in a social motivation test [70–72] but explained only 1.6% to 4.9% of the phenotypic variance [70]. In rodents, Flint [19] also showed that most behaviors were influenced by QTLs with small effects, that each contribute to less than 10% of the phenotypic variance of a behavioral trait.

### Genes underlying behavioral reactivity traits

Despite the preliminary nature of our findings and the need for further studies in order to validate the QTL effects found in our study before being effectively able to conduct a candidate gene approach, it is worth commenting on the genes that may underlie the most relevant QTL effects described here. We used bioinformatics tools [73, 74] to search for possible coincidences between our QTL regions (based either on flanking intervals or fine location) and the location of genes that have already been associated with behavioral traits in a variety of mammals. The QTL region on chromosome 16 (39.5-49.8 Mb) associated with both high bleats in response to social separation and flight distance from a human appeared as one of the most interesting regions in which to search for candidate genes. Interestingly, this region coincides with a QTL region found in cattle on chromosome 20 for the flight distance from a feeder [41]. Several coding genes are located close to the fine location of the QTL in this specific region of OAR16 that could be involved in behavioral responses, although no scientific evidence has yet suggested their involvement in regulating behavioral responses. Among these genes, the gene NPR3 (Atrial Natriuretic Peptide Receptor 3, 44.2 Mb) codes for a protein that acts as a clearance receptor for brain natriuretic peptides and helps to regulate blood pressure. Several candidate genes associated with behavioral traits are also located close to or within the QTL region on chromosome 21 (37.9-48.4 Mb) found in our study to be associated with vocalization behaviors (both low and high bleats). This is the case of the gene CHRM1 (cholinergic receptor muscarinic 1, 40.6 Mb). This gene has been described to play a key role in locomotion, cognition and nervous system development [75]. Interestingly, a QTL for a vocalization behavior has been found in cattle on chromosome 29 in a region of conserved synteny [41] but without an identified candidate gene. On chromosome 12, the QTL mapped at 56.3 Mb and associated with low bleats overlaps with the gene ASTN1 (Astrotactin 1, 56.0 Mb). This gene is associated with substance abuse, bipolar disorder and schizophrenia in humans [76]. Finally, the QTL associated with locomotion in response to social isolation on OAR19 (18.9 Mb) maps within the gene GRM7 (Glutamate receptor metabotropic 7, 18.0 Mb) known to be involved in schizophrenia [77]. In addition, this QTL is close to the gene encoding the oxytocin receptor (OXTR, 17.6 Mb) which is associated with social [78] and maternal behaviors [79, 80].

## Conclusion

The work reported here is the first SNP-based QTL detection study for behavioral traits in sheep. We reported various QTLs with low to moderate effects. Five main QTL regions associated with social reactivity and reactivity to humans were identified on OAR12, 16, 19, 21 and 23. These QTL regions contain interesting candidate genes previously described to be associated with various social and/or emotional behaviors in mammals. Ultimately, the identification of genes underlying the behavioral reactivity assessed in our study will provide opportunities for deeper understanding the genetic components involved and will contribute to the general understanding of adaptive traits in animals. If these SNP-trait associations are confirmed, these SNP markers could potentially be used to improve behavioral adaptation in sheep.

### Availability of supporting data and requirements

The data sets supporting the results of this article are included within the article and its additional files. Programs, scripts and other information used to set up the analyses can be obtained from the corresponding author upon request.

## Electronic supplementary material

Additional file 1: Table S6: Fixed effects and behavioral traits measured in lambs submitted to behavioral tests. (CSV 230 KB)

Additional file 2: Table S4: Factor loadings for the first four extracted factors for each variable from the three behavioral tests included and the variance explained by each factor. This file contains factor loadings for the first four extracted factors for each variable resulting from the factor analysis on the 15 behavioral traits used in this study. (DOCX 15 KB)

Additional file 3: Table S1: Complete list of QTLs detected by linkage analysis. This file contains a table that lists all the significant QTLs found by linkage analysis for the 16 traits and provides the significance, position of maximum likelihood ratio test, confidence interval and average QTL effect. (DOCX 21 KB)

Additional file 4: Table S2: Complete list of QTLs detected by GWAS. This file contains a table that lists all the significant QTLs found by association analysis for the 16 traits and provides the significance, position of maximum likelihood ratio test, confidence interval, number of haplotypes and the name of flanking markers. (DOCX 43 KB)

Additional file 5: Table S3: Complete list of QTLs detected by LDLA analysis. This file contains a table that lists all the significant QTLs found by joint linkage and association analysis for the 16 traits and provides the significance, position of maximum likelihood ratio test, confidence interval, number of haplotypes and the name of flanking markers. (DOCX 24 KB)

Additional file 6: Table S5: Estimates of heritabilities and variances ± S.E for behavioral and physiological traits. This file contains heritability of traits analyzed in this study, proportion of phenotypic variance attributed to maternal, litter and residual effects and the total phenotypic variance for each trait. (DOCX 18 KB)
