# Supplementary material for: Identification of QTLs for behavioral reactivity to social separation and humans in sheep using the OvineSNP50 BeadChip
Source: BMC Genomics. 2014 Sep 9;15(1):778. doi: 10.1186/1471-2164-15-778 (PMC4171556; doi:10.1186/1471-2164-15-778)
Supplement: Supplementary file 2 — Additional file 2: Table S4: Factor loadings for the first four extracted factors for each variable from the three behavioral tests included and the variance explained by each factor. This file contains factor loadings for the first four extracted factors for each variable resulting from the factor analysis on the 15 behavioral traits used in this study. (DOCX 15 KB) [file 12864_2014_6464_MOESM2_ESM.docx]

**Additional file 2: Table S4 Factor loadings for the first four extracted factors for each variable from the three behavioral tests included and the variance explained by each factor.**

| **Original measures** | **Factor 1** | **Factor 2** | **Factor 3** | **Factor 4** | **Communality** |
| --- | --- | --- | --- | --- | --- |
| AT1-LOCOM  AT2-LOCOM  CT1-LOCOM  IBT-LOCOM  AT1-HBLEAT  AT2-HBLEAT  CT1-HBLEAT  IBT-HBLEAT  AT1-LBLEAT  AT2-LBLEAT  CT1-LBLEAT  AT1-VIGIL  AT2-PROX  CT2-DIST  CT2-SEEN  Variance (%) | 0.08  0.14  0.10  0.02  **0.80**  **0.73**  **0.81**  **0.77**  -0.44  -0.02  0.07  0.02  0.15  -0.07  0.05  21.0 | 0.00  0.01  -0.39  -0.16  0.16  -0.17  0.16  0.10  -0.02  0.00  -0.05  -0.09  **0.56**  **-0.85**  **0.86**  14.6 | **0.83**  **0.41**  0.34  **0.44**  0.29  0.04  0.10  0.01  0.05  0.03  0.00  **-0.78**  0.28  0.14  -0.07  12.3 | 0.05  0.02  0.12  -0.01  0.06  -0.15  -0.22  0.11  **0.69**  **0.74**  **0.82**  0.01  0.06  0.03  -0.03  9.2 | 70.4  18.7  29.7  22.3  75.5  59.7  73.6  62.1  66.7  55.0  68.6  62.3  42.1  75.3  74.1 |

Bold values have Eigen value > 0.40. Communality gives proportion of the variability of a behavioral variable explained by the four factors.
