# Supplementary material for: Identification of QTLs for behavioral reactivity to social separation and humans in sheep using the OvineSNP50 BeadChip
Source: BMC Genomics. 2014 Sep 9;15(1):778. doi: 10.1186/1471-2164-15-778 (PMC4171556; doi:10.1186/1471-2164-15-778)
Supplement: Supplementary file 3 — Additional file 3: Table S1: Complete list of QTLs detected by linkage analysis. This file contains a table that lists all the significant QTLs found by linkage analysis for the 16 traits and provides the significance, position of maximum likelihood ratio test, confidence interval and average QTL effect. (DOCX 21 KB) [file 12864_2014_6464_MOESM3_ESM.docx]

**Additional file 3: Table S1** Complete list of QTL detected in linkage analysis

| **OAR** | **Trait** | **Significance^1^** | **level** | **Position^2^ (Mb)** | **Confidence Interval** | **Average QTL Effect^3^** | **QTL heritability (%)** |
| --- | --- | --- | --- | --- | --- | --- | --- |
| 2 | AT1-HBLEAT | * | CW | 244.8 | 241.2 – 248.4 | 0.24 | 2.63 |
| 2 | AT2-HBLEAT | * | CW | 218.9 | 216.8 – 219.9 | 0.26 | 3.79 |
| 2 | IBT-LOCOM | * | CW | 250.1 | 249.2 – 254.1 | 0.22 | 3.23 |
| 4 | AT2-PROX | * | CW | 91.3 | 90.1 – 104.5 | 0.19 | 3.14 |
| 5 | ISO_HBLEAT | ** | CW | 96.8 | 92.8 – 97.3 | 0.22 | 3.36 |
| 5 | AT1-HBLEAT | * | CW | 5.7 | 5.0 – 7.6 | 0.25 | 2.70 |
| 5 | CT1-HBLEAT | * | GW | 93.7 | 92.0 – 95.4 | 0.25 | 3.39 |
| 5 | IBT-HBLEAT | * | GW | 52.6 | 47.0 – 55.0 | 0.22 | 3.36 |
| 5 | FACTOR1 | ** | CW | 93.8 | 92.0 - 95.3 | 0.26 | 3.00 |
| 6 | FACTOR2 | ** | CW | 111.7 | 111.6 - 111.8 | 0.22 | 3.33 |
| 10 | AT2-LOCOM | ** | CW | 16.1 | 14.2 – 17.8 | 0.20 | 3.08 |
| 12 | ISO_LBLEAT | * | GW | 67.8 | 65.6 – 70.9 | 0.22 | 3.65 |
| 12 | AT1-LBLEAT | * | CW | 70.4 | 66.4 – 71.2 | 0.27 | 3.26 |
| 12 | CT1-LBLEAT | ** | CW | 27.5 (68.4) | 26.1 – 29.1 | 0.21 | 3.29 |
| 12 | CT2-DIST | * | CW | 33.1 | 29.7 – 39.4 | 0.23 | 2.97 |
| 12 | FACTOR4 | * | CW | 69.0 | 63.7 - 71.1 | 0.20 | 2.62 |
| 13 | ISO_HBLEAT | * | CW | 39.7 | 37.5 – 43.9 | 0.22 | 2.79 |
| 13 | CT1-HBLEAT | ** | GW | 41.4 | 40.5 – 44.0 | 0.27 | 4.00 |
| 13 | FACTOR1 | * | CW | 41.4 | 37.1 - 43.1 | 0.16 | 1.96 |
| 14 | AT1-LOCOM | * | CW | 67.3 | 60.3 – 69.3 | 0.25 | 2.86 |
| 16 | ISO_HBLEAT | ** | CW | 43.7 | 41.3 – 49.8 | 0.24 | 3.28 |
| 16 | AT1-HBLEAT | * | CW | 47.7 | 39.5 – 49.8 | 0.21 | 2.41 |
| 16 | AT2-HBLEAT | *** | GW | 45.1 | 41.7 – 46.6 | 0.30 | 6.18 |
| 16 | IBT-HBLEAT | * | CW | 45.8 | 42.2 – 48.8 | 0.25 | 3.38 |
| 16 | HUMAPPRO | * | CW | 48.0 (34.4) | 42.4 – 55.7 | 0.22 | 2.76 |
| 16 | CT2-DIST | ** | CW | 34.4 (48.0) | 33.0 – 36.8 | 0.24 | 3.11 |
| 16 | FACTOR1 | *** | GW | 45.2 | 41.9 - 48.1 | 0.24 | 3.84 |
| 16 | FACTOR2 | * | CW | 46.1 | 39.0 - 57.7 | 0.21 | 2.01 |
| 17 | CT1-HBLEAT | ** | CW | 39.0 | 35.1 – 42.3 | 0.19 | 3.24 |
| 17 | ISO_LBLEAT | * | GW | 33.3 | 30.5 – 36.8 | 0.26 | 2.51 |
| 17 | AT1-LBLEAT | * | CW | 33.4 | 30.5 – 42.3 | 0.18 | 2.57 |
| 17 | CT1-LBLEAT | * | CW | 52.7 | 47.6 – 55.2 | 0.21 | 2.56 |
| 17 | IBT-LOCOM | * | CW | 66.4 | 64.7 – 72.0 | 0.21 | 3.03 |
| 19 | CORT | * | CW | 41.0 | 38.9 – 43.2 | 0.27 | 4.02 |
| 20 | IBT-LOCOM | ** | CW | 38.3 | 36.9 – 43.2 | 0.26 | 3.77 |
| 21 | AT2-HBLEAT | * | CW | 36.0 | 14.7 – 37.1 | 0.19 | 2.73 |
| 21 | CT1-HBLEAT | * | CW | 36.9 | 33.3 – 46.1 | 0.20 | 2.57 |
| 21 | ISO_LBLEAT | ** | GW | 39.3 | 38.1 – 40.05 | 0.26 | 4.37 |
| 21 | AT1-LBLEAT | ** | CW | 39.3 | 37.9 – 41.6 | 0.22 | 3.34 |
| 21 | CT1-LBLEAT | * | GW | 47.3 | 46.5 – 48.4 | 0.26 | 3.80 |
| 21 | AT1-VIGIL | * | CW | 10.8 | 9.6 – 11.7 | 0.21 | 3.01 |
| 21 | FACTOR3 | * | CW | 10.9 | 10.2 - 14.2 | 0.20 | 2.54 |
| 21 | FACTOR4 | *** | GW | 47.6 | 45.9 - 48.5 | 0.27 | 3.99 |
| 22 | AT1-VIGIL | * | CW | 47.1 | 46.0 – 50.1 | 0.21 | 2.94 |
| 23 | IBT-LOCOM | * | CW | 56.7 | 55.8 – 59.2 | 0.19 | 2.31 |
| 24 | CT1-LBLEAT | ** | CW | 9.8 | 9.1 – 11.7 | 0.19 | 3.21 |
| 24 | AT2-LOCOM | * | CW | 41.8 | 41.4 – 42.7 | 0.22 | 3.30 |
| 24 | FACTOR4 | * | CW | 10.1 | 9.6 - 10.7 | 0.29 | 5.54 |
| 26 | FACTOR2 | * | CW | 42.45 | 41.9 - 44.7 | 0.21 | 2.01 |

^1^: *, p < 5%; **, p < 1%; ***, p < 0.1%. ^2^: position of a second significant QTL indicated between parentheses. ^3^: average QTL effect given in phenotypic standard deviation. CW, chromosome wide; GW, genome wide.
