# Supplementary material for: Identification of QTLs for behavioral reactivity to social separation and humans in sheep using the OvineSNP50 BeadChip
Source: BMC Genomics. 2014 Sep 9;15(1):778. doi: 10.1186/1471-2164-15-778 (PMC4171556; doi:10.1186/1471-2164-15-778)
Supplement: Supplementary file 4 — Additional file 4: Table S2: Complete list of QTLs detected by GWAS. This file contains a table that lists all the significant QTLs found by association analysis for the 16 traits and provides the significance, position of maximum likelihood ratio test, confidence interval, number of haplotypes and the name of flanking markers. (DOCX 43 KB) [file 12864_2014_6464_MOESM4_ESM.docx]

**Additional file 4: Table S2** Complete list of QTL detected in GWAS

| **OAR** | **Trait** | **Signifi-cance^1^** | **le-vel** | **Pos^2^ (Mb)** | **Confidence Interval** | **No. haplotypes** | **Flanking markers^3^** | | **Haplotype with the highest effect** | | | **haplotype with the lowest effect** | | |
| --- | --- | --- | --- | --- | --- | --- | --- | --- | --- | --- | --- | --- | --- | --- |
|  |  |  |  |  |  |  |  |  | **Freq.** | **Effect** | **Var.^4^ (%)** | **Freq.** | **Effect** | **Var.^4^ (%)** |
| 1 | IBT-HBLEAT | * | CW | 264.8 | 264.7 - 266.5 | 14 | s60324.1 | OAR1_265023775.1 | 0.01 | 0.618 | 0.56 | 0.11 | -0.234 | 0.80 |
| 2 | AT1-LBLEAT | * | CW | 204.7 | 204.6 - 204.9 | 12 | OAR2_204684212.1 | OAR2_204712172.1 | 0.03 | 0.174 | 1.26 | 0.02 | -0.042 | 0.10 |
| 2 | AT2-LBLEAT | * | CW | 164.6 | 151.4 - 165.0 | 8 | OAR2_164592801.1 | OAR2_164635032.1 | 0.01 | 0.434 | 4.14 | 0.08 | -0.052 | 0.44 |
| 2 | AT2-LOCOM | * | CW | 181.7 | 172.6 - 181.8 | 11 | OAR2_181662730.1 | OAR2_181764446.1 | 0.03 | 4.203 | 5.88 | 0.03 | -0.908 | 0.27 |
| 2 | ISO_HBLEAT | ** | CW | 143.8 | 143.7 - 151.1 | 11 | s01640.1 | OAR2_143893183.1 | 0.02 | 0.71 | 0.98 | 0.1 | -0.32 | 0.91 |
| 2 | ISO_LOCOM | * | CW | 210.2 | 203.2 - 210.3 | 9 | OAR2_210121419.1 | OAR2_210205525.1 | 0.31 | 0.355 | 3.85 | 0.08 | -0.372 | 1.46 |
| 2 | FACTOR1 | * | GW | 143.8 | 143.7 - 143.9 | 11 | s01640.1 | OAR2_143893183.1 | 0.02 | 0.519 | 1.11 | 0.1 | -0.204 | 0.79 |
| 3 | AT2-LBLEAT | * | CW | 24.1 | 24.0 - 41.1 | 11 | OAR3_23863789.1 | OAR3_24047322.1 | 0.07 | 0.151 | 3.30 | 0.01 | -0.138 | 0.42 |
| 3 | AT2-LOCOM | * | CW | 238.9 | 238.8 - 239.0 | 11 | s32797.1 | s53303.1 | 0.04 | 4.298 | 8.11 | 0.07 | -0.362 | 0.10 |
| 3 | FACTOR4 | ** | CW | 136.8 | 136.7 - 136.9 | 9 | OAR3_136802294.1 | OAR3_136865536.1 | 0.01 | 0.848 | 1.27 | 0.04 | -0.366 | 0.92 |
| 4 | IBT-LOCOM | * | CW | 9.7 | 6.3 - 23.4 | 8 | OAR4_9681181_X.1 | OAR4_9720138_X.1 | 0.02 | 0.716 | 0.83 | 0.14 | -0.061 | 0.10 |
| 4 | FACTOR1 | * | CW | 59.7 | 59.6 - 59.8 | 12 | OAR4_59677745.1 | OAR4_59725502.1 | 0.09 | 0.531 | 4.86 | 0.04 | -0.534 | 2.31 |
| 5 | AT1-LBLEAT | ** | CW | 80.3 | 74.3 - 80.4 | 10 | OAR5_80246247.1 | OAR5_80304302.1 | 0.13 | 0.154 | 3.83 | 0.02 | -0.075 | 0.16 |
| 5 | AT2-LOCOM | * | CW | 49.2 | 49.1 - 49.3 | 12 | OAR5_49181962.1 | OAR5_49283556.1 | 0.05 | 2.935 | 4.68 | 0.05 | -0.602 | 0.20 |
| 5 | FACTOR1 | * | CW | 92.1 | 92.0 - 92.2 | 11 | OAR5_92064206.1 | OAR5_92169254.1 | 0.02 | 0.521 | 1.12 | 0.03 | -0.51 | 1.59 |
| 5 | FACTOR3 | * | CW | 63.8 | 62.2 - 77.5 | 8 | OAR5_63727768.1 | OAR5_63804089.1 | 0.09 | 0.492 | 4.05 | 0.27 | -0.053 | 0.11 |
| 6 | AT1-HBLEAT | * | CW | 106.9 | 99.3 - 108.8 | 11 | s05095.1 | s25229.1 | 0.05 | 0.289 | 0.47 | 0.06 | -0.525 | 1.84 |
| 6 | CT1-HBLEAT | * | CW | 106.9 | 106.8 - 107.0 | 11 | s05095.1 | s25229.1 | 0.05 | 0.273 | 0.58 | 0.04 | -0.614 | 2.35 |
| 6 | CT2-SEEN | * | CW | 103.2 | 100.1 - 103.3 | 10 | OAR6_103085812.1 | OAR6_103247069.1 | 0.02 | 8.352 | 5.69 | 0.05 | -2.151 | 0.91 |
| 6 | ISO_HBLEAT | * | CW | 106.9 | 106.8 - 107.0 | 11 | s05095.1 | s25229.1 | 0.05 | 0.457 | 0.98 | 0.04 | -0.765 | 2.23 |
| 6 | FACTOR2 | ** | CW | 110.0 | 100.1 - 113.2 | 9 | s29906.1 | OAR6_110056838.1 | 0.12 | -0.034 | 0.10 | 0.02 | -0.646 | 1.80 |
| 7 | ISO_LBLEAT | ** | CW | 52.1 | 43.5 - 52.2 | 9 | OAR7_52124140.1 | OAR7_52157129.1 | 0.02 | 1.186 | 3.45 | 0.19 | -0.057 | 0.10 |
| 8 | AT1-HBLEAT | * | CW | 71.5 | 71.4 - 80.3 | 11 | s47800.1 | OAR8_71548278.1 | 0.02 | 0.251 | 0.15 | 0.09 | -0.556 | 3.00 |
| 8 | AT1-LBLEAT | * | GW | 69.1 | 60.9 - 69.2 | 10 | OAR8_69105959.1 | OAR8_69151787.1 | 0.01 | 0.253 | 0.91 | 0.09 | -0.063 | 0.46 |
| 8 | AT2-LOCOM | * | CW | 66.4 | 63.5 - 66.5 | 11 | OAR8_66387084.1 | OAR8_66520139.1 | 0.15 | 1.446 | 3.05 | 0.19 | -0.157 | 0.10 |
| 9 | AT2-HBLEAT | * | CW | 84.3 | 84.2 - 84.4 | 10 | s65296.1 | OAR9_84346672.1 | 0.07 | 0.413 | 1.67 | 0.02 | -0.498 | 0.73 |
| 9 | FACTOR1 | * | CW | 84.3 | 83.9 - 84.4 | 10 | s65296.1 | OAR9_84346672.1 | 0.03 | 0.283 | 0.49 | 0.05 | -0.183 | 0.33 |
| 10 | AT1-LBLEAT | * | CW | 93.9 | 93.8 - 94.0 | 8 | s69331.1 | DU455689_544.1 | 0.02 | 0.503 | 7.08 | 0.02 | -0.076 | 0.16 |
| 10 | CT2-DIST | * | CW | 54.3 | 54.0 - 54.4 | 12 | OAR10_54264286.1 | OAR10_54347258.1 | 0.02 | 0.526 | 0.82 | 0.01 | -0.365 | 0.20 |
| 10 | IBT-HBLEAT | * | CW | 68.1 | 65.7 - 68.2 | 11 | OAR10_68107947.1 | OAR10_68142866.1 | 0.12 | 0.589 | 5.47 | 0.11 | -0.199 | 0.58 |
| 10 | ISO_HBLEAT | * | CW | 65.7 | 64.7 - 68.1 | 14 | OAR10_65705353.1 | OAR10_65756510.1 | 0.05 | 0.428 | 0.86 | 0.11 | -0.588 | 3.35 |
| 12 | AT1-LBLEAT | * | CW | 56.3 | 53.2 - 80.5 | 13 | s63508.1 | OAR12_56302501_X.1 | 0.02 | 0.383 | 4.11 | 0.16 | 0.013 | 0.10 |
| 12 | AT2-LBLEAT | * | GW | 56.3 | 56.1 - 61.6 | 13 | s63508.1 | OAR12_56302501_X.1 | 0.02 | 0.357 | 5.55 | 0.12 | -0.034 | 0.27 |
| 12 | CT2-DIST | ** | CW | 32.6 | 25.7 - 32.7 | 10 | OAR12_32559849.1 | OAR12_32617508.1 | 0.05 | 0.539 | 2.09 | 0.12 | -0.195 | 0.61 |
| 12 | CT2-SEEN | * | CW | 39.7 | 25.5 - 39.8 | 10 | OAR12_39622345.1 | OAR12_39680259.1 | 0.03 | 5.226 | 3.31 | 0.04 | -1.488 | 0.35 |
| 12 | HUMAPPRO | * | CW | 39.7 | 25.2 - 39.8 | 10 | OAR12_39622345.1 | OAR12_39680259.1 | 0.03 | 0.861 | 2.57 | 0.04 | -0.471 | 1.01 |
| 12 | IBT-HBLEAT | * | CW | 85.2 | 79.8 - 85.3 | 9 | s72376.1 | s33393.1 | 0.09 | 0.461 | 2.60 | 0.03 | -0.238 | 0.25 |
| 12 | ISO_LBLEAT | ** | GW | 56.2 | 56.1 - 56.3 | 13 | s63508.1 | OAR12_56302501_X.1 | 0.02 | 1.344 | 4.43 | 0.16 | 0.01 | 0.10 |
| 12 | FACTOR4 | ** | GW | 56.3 | 56.2 - 56.4 | 13 | s63508.1 | OAR12_56302501_X.1 | 0.02 | 1.306 | 5.97 | 0.03 | -0.071 | 0.10 |
| 13 | AT1-HBLEAT | * | CW | 34.0 | 31.4 - 35.8 | 10 | OAR13_33983722.1 | OAR13_34056956.1 | 0.1 | 0.236 | 0.59 | 0.01 | -1.456 | 2.48 |
| 13 | AT2-HBLEAT | * | CW | 2.8 | 2.4 - 8.6 | 10 | s26388.1 | OAR13_2846634_X.1 | 0.06 | 0.469 | 1.87 | 0.37 | -0.096 | 0.32 |
| 13 | CT1-LBLEAT | * | CW | 20.5 | 20.4 - 29.3 | 12 | s30126.1 | OAR13_20537279.1 | 0.02 | 0.265 | 3.06 | 0.04 | -0.128 | 1.40 |
| 13 | ISO_HBLEAT | * | CW | 45.6 | 34.0 - 45.7 | 13 | s73104.1 | s43103.1 | 0.03 | 0.651 | 1.22 | 0.01 | -1.732 | 2.94 |
| 13 | ISO_LBLEAT | * | CW | 63.0 | 62.9 - 63.2 | 11 | OAR13_62949442.1 | s33377.1 | 0.07 | 0.694 | 3.92 | 0.02 | -0.749 | 1.37 |
| 13 | FACTOR1 | ** | CW | 45.6 | 39.3 - 51.2 | 13 | s73104.1 | s43103.1 | 0.03 | 0.538 | 1.77 | 0.01 | -1.317 | 3.62 |
| 13 | FACTOR4 | ** | CW | 64.3 | 62.5 - 64.4 | 9 | s00952.1 | s70439.1 | 0.05 | 0.75 | 4.77 | 0.05 | -0.317 | 0.85 |
| 14 | AT2-LBLEAT | * | CW | 67.5 | 67.4 - 67.7 | 9 | OAR14_67510494.1 | OAR14_67561824.1 | 0.08 | 0.051 | 0.43 | 0.01 | -0.141 | 0.44 |
| 15 | AT1-HBLEAT | * | CW | 13.2 | 13.1 - 20.5 | 10 | OAR15_13151004.1 | OAR15_13214168.1 | 0.48 | 0.101 | 0.30 | 0.05 | -0.951 | 5.08 |
| 15 | AT1-LBLEAT | *** | CW | 74.8 | 74.7 - 74.9 | 10 | OAR15_74759937.1 | OAR15_74809345.1 | 0.02 | 0.289 | 2.34 | 0.03 | -0.023 | 0.10 |
| 15 | FACTOR2 | * | CW | 4.0 | 3.9 - 8.7 | 9 | OAR15_3963856.1 | OAR15_4058002.1 | 0.23 | 0.119 | 0.55 | 0.03 | -0.421 | 1.13 |
| 16 | AT1-HBLEAT | * | CW | 46.3 | 42.7 - 46.4 | 13 | OAR16_46290531.1 | OAR16_46325523.1 | 0.06 | 0.328 | 0.72 | 0.08 | -0.45 | 1.76 |
| 16 | AT2-HBLEAT | ** | GW | 44.3 (20.1) | 44.0 - 45.7 | 14 | OAR16_44325630.1 | s23014.1 | 0.01 | 0.812 | 0.98 | 0.01 | -0.513 | 0.39 |
| 16 | AT2-LBLEAT | * | CW | 28.9 | 21.0 - 30.2 | 9 | OAR16_28856625.1 | s69560.1 | 0.01 | 0.564 | 7.00 | 0.36 | -0.025 | 0.32 |
| 16 | CT2-DIST | * | CW | 45.4 | 45.3 - 46.3 | 12 | OAR16_45398511.1 | s17055.1 | 0.2 | 0.291 | 2.05 | 0.06 | -0.353 | 1.06 |
| 16 | IBT-HBLEAT | * | CW | 46.3 | 44.4 - 46.8 | 13 | OAR16_46290531.1 | OAR16_46325523.1 | 0.06 | 0.544 | 2.49 | 0.04 | -0.193 | 0.21 |
| 16 | IBT-LOCOM | * | CW | 63.5 | 62.3 - 66.6 | 6 | OAR16_63465429.1 | OAR16_63522482.1 | 0.02 | 1.824 | 5.37 | 0.06 | -0.125 | 0.10 |
| 16 | ISO_HBLEAT | * | GW | 43.9 | 43.5 - 46.7 | 11 | OAR16_43833978.1 | OAR16_43916302.1 | 0.01 | 0.742 | 0.54 | 0.1 | -0.27 | 0.65 |
| 16 | FACTOR1 | *** | GW | 43.9 | 43.8 - 46.3 | 11 | OAR16_43833978.1 | OAR16_43916302.1 | 0.01 | 0.636 | 0.84 | 0.16 | -0.177 | 0.89 |
| 17 | AT1-HBLEAT | * | CW | 12.7 | 12.6 - 15.0 | 12 | s42157.1 | OAR17_12809597.1 | 0.02 | 0.489 | 0.55 | 0.04 | -0.771 | 2.70 |
| 17 | CT2-DIST | * | CW | 11.1 | 11.0 - 29.8 | 11 | OAR17_11099220.1 | DU324670_456.1 | 0.04 | 0.89 | 4.61 | 0.01 | -0.622 | 0.58 |
| 17 | IBT-LOCOM | * | CW | 40.6 | 22.7 - 40.7 | 9 | OAR17_40579701.1 | OAR17_40700344.1 | 0.02 | 1.679 | 4.55 | 0.02 | -0.387 | 0.24 |
| 17 | ISO_HBLEAT | * | CW | 12.7 | 12.6 - 15.0 | 12 | s42157.1 | OAR17_12809597.1 | 0.02 | 1.213 | 2.86 | 0.02 | -0.532 | 0.55 |
| 17 | FACTOR2 | *** | CW | 5.9 | 5.8 - 7.3 | 11 | OAR17_5938733.1 | OAR17_5979442.1 | 0.01 | 0.836 | 1.52 | 0.09 | -0.436 | 3.42 |
| 18 | AT2-HBLEAT | * | CW | 45.2 | 45.1 - 58.5 | 14 | OAR18_45139986.1 | s59000.1 | 0.03 | 0.386 | 0.65 | 0.01 | -0.999 | 1.49 |
| 18 | CT2-SEEN | * | CW | 7.7 | 7.6 - 7.8 | 11 | s03865.1 | s48460.1 | 0.01 | 9.352 | 3.60 | 0.01 | -2.675 | 0.29 |
| 18 | HUMAPPRO | * | CW | 7.7 | 7.6 - 7.8 | 11 | s03865.1 | s48460.1 | 0.01 | 2.576 | 7.82 | 0.01 | -0.647 | 0.49 |
| 18 | FACTOR2 | * | CW | 29.1 | 26.8 - 38.5 | 11 | DU304162_428.1 | OAR18_29143813.1 | 0.16 | 0.175 | 0.90 | 0.02 | -0.517 | 1.15 |
| 19 | AT1-LBLEAT | * | CW | 49.3 | 49.2 - 49.5 | 12 | s58019.1 | OAR19_49422424.1 | 0.01 | 0.331 | 1.55 | 0.05 | -0.051 | 0.18 |
| 19 | AT2-LOCOM | *** | GW | 18.9 | 18.8 - 19.0 | 9 | OAR19_18888791.1 | s24963.1 | 0.01 | 6.979 | 5.51 | 0.06 | -1.058 | 0.72 |
| 19 | IBT-LOCOM | * | CW | 18.0 | 17.9 - 18.1 | 10 | s56027.1 | OAR19_18010247.1 | 0.08 | 0.509 | 1.57 | 0.08 | -0.139 | 0.12 |
| 19 | CORT | * | CW | 39.1 | 39.0 - 41.8 | 7 | OAR19_39084409.1 | OAR19_39123291.1 | 0.08 | 0.122 | 7.30 | 0.11 | -0,003 | 0.10 |
| 19 | FACTOR3 | * | CW | 13.9 | 13.5 - 14.7 | 11 | OAR19_13792929.1 | s27730.1 | 0.03 | 0.484 | 1.39 | 0.03 | -0.282 | 0.47 |
| 20 | AT1-VIGIL | * | CW | 26.4 | 25.6 - 26.5 | 12 | OAR20_26411399.1 | s18641.1 | 0.03 | 2.655 | 0.46 | 0.05 | -3.279 | 1.15 |
| 20 | CT2-SEEN | ** | CW | 7.6 | 7.5 - 7.7 | 10 | OAR20_7626319.1 | OAR20_7794638.1 | 0.08 | 2.824 | 2.44 | 0.03 | -2.02 | 0.49 |
| 21 | AT1-HBLEAT | * | CW | 43.1 | 37.7 - 43.2 | 10 | OAR21_43118557.1 | s61819.1 | 0.02 | 0.732 | 1.24 | 0.09 | -0.378 | 1.38 |
| 21 | AT1-LBLEAT | * | GW | 7.6 | 7.2 - 7.7 | 14 | s54902.1 | OAR21_7730122.1 | 0.01 | 0.672 | 6.39 | 0.08 | -0.095 | 0.95 |
| 21 | AT2-HBLEAT | * | CW | 38.0 | 25.3 - 45.1 | 9 | OAR21_38037300_X.1 | OAR21_38087037.1 | 0.01 | 1.041 | 1.61 | 0.04 | -0.385 | 0.86 |
| 21 | IBT-LOCOM | * | CW | 7.7 | 7.6 - 23.7 | 11 | OAR21_7730122.1 | s10072.1 | 0.03 | 0.914 | 2.00 | 0.03 | -0.378 | 0.34 |
| 21 | ISO_HBLEAT | * | CW | 43.1 | 36.6 - 45.1 | 10 | OAR21_43118557.1 | s61819.1 | 0.02 | 0.758 | 1.11 | 0.09 | -0.281 | 0.64 |
| 21 | FACTOR1 | ** | CW | 38.0 | 36.6 - 40.4 | 9 | OAR21_38037300_X.1 | OAR21_38087037.1 | 0.01 | 0.683 | 0.97 | 0.04 | -0.121 | 0.12 |
| 23 | AT2-HBLEAT | * | CW | 34.4 | 33.6 - 41.8 | 11 | OAR23_34225387.1 | OAR23_34401277.1 | 0.01 | 0.454 | 0.31 | 0.01 | -0.616 | 0.56 |
| 23 | AT2-LOCOM | * | GW | 54.1 | 51.6 - 54.2 | 10 | OAR23_54064411.1 | s31567.1 | 0.03 | 2.985 | 2.96 | 0.08 | -0.589 | 0.29 |
| 23 | CT1-LBLEAT | * | CW | 50.2 | 45.4 - 58.7 | 11 | s47100.1 | OAR23_50255468.1 | 0.15 | 0.13 | 4.79 | 0.01 | -0.101 | 0.22 |
| 23 | CT2-DIST | * | CW | 32.2 | 28.9 - 32.4 | 12 | OAR23_32137172.1 | s55273.1 | 0.02 | 0.981 | 2.86 | 0.08 | -0.202 | 0.46 |
| 23 | CT2-SEEN | ** | CW | 32.3 | 27.9 - 32.4 | 10 | s55273.1 | s74857.1 | 0.01 | 2.432 | 0.24 | 0.02 | -5.799 | 2.74 |
| 23 | HUMAPPRO | ** | CW | 32.2 | 28.9 - 32.3 | 12 | OAR23_32137172.1 | s55273.1 | 0.08 | 0.296 | 0.77 | 0.02 | -1.064 | 2.64 |
| 23 | ISO_LBLEAT | * | CW | 50.2 | 43.5 - 52.7 | 11 | s47100.1 | OAR23_50255468.1 | 0.15 | 0.542 | 4.68 | 0.01 | -0.39 | 0.19 |
| 23 | FACTOR2 | * | GW | 32.2 | 32.1 - 32.4 | 12 | OAR23_32137172.1 | s55273.1 | 0.08 | 0.186 | 0.56 | 0.02 | -0.951 | 3.90 |
| 23 | FACTOR3 | * | CW | 3.2 | 3.1 - 18.1 | 11 | OAR23_3143631.1 | OAR23_3266641.1 | 0.08 | 0.405 | 2.46 | 0.07 | -0.252 | 0.84 |
| 24 | AT1-LBLEAT | * | CW | 8.2 | 5.5 - 8.3 | 11 | s04466.1 | s56297.1 | 0.01 | 0.322 | 1.47 | 0.02 | -0.208 | 1.21 |
| 24 | ISO_LBLEAT | * | CW | 8.2 | 6.8 - 8.3 | 11 | s04466.1 | s56297.1 | 0.01 | 1.038 | 1.33 | 0.02 | -0.619 | 0.94 |
| 25 | AT1-HBLEAT | * | CW | 29.0 | 28.9 - 29.1 | 9 | s13005.1 | s53175.1 | 0.12 | 0.252 | 0.79 | 0.01 | -1.801 | 3.80 |
| 25 | FACTOR3 | * | CW | 31.9 | 31.8 - 32.0 | 11 | OAR25_31929813.1 | s60999.1 | 0.06 | 0.532 | 3.26 | 0.02 | -0.22 | 0.19 |
| 26 | CT1-HBLEAT | * | CW | 45.4 | 45.3 - 46.8 | 13 | s31164.1 | s16475.1 | 0.01 | 0.378 | 0.23 | 0.09 | -0.261 | 0.91 |
| 26 | CT2-DIST | * | CW | 5.1 | 5.0 - 5.3 | 11 | OAR26_5062133.1 | DU381007_457.1 | 0.03 | 0.768 | 2.60 | 0.02 | -0.747 | 1.66 |
| 26 | IBT-HBLEAT | * | CW | 45.4 | 45.3 - 45.6 | 13 | s31164.1 | s16475.1 | 0.01 | 0.769 | 0.87 | 0.06 | -0.194 | 0.32 |
| 26 | ISO_HBLEAT | ** | CW | 45.4 | 45.3 - 46.8 | 13 | s31164.1 | s16475.1 | 0.01 | 0.667 | 0.44 | 0.06 | -0.379 | 0.80 |
| 26 | FACTOR1 | ** | CW | 6.4 | 4.5 - 9.5 | 10 | s58657.1 | DU481531_204.1 | 0.15 | 0.322 | 2.78 | 0.01 | -0.707 | 1.04 |
| 26 | FACTOR2 | * | CW | 41.2 | 39.8 - 47.6 | 10 | OAR26_41166096.1 | s07654.1 | 0.07 | 0.112 | 0.18 | 0.15 | -0.368 | 3.79 |
| 26 | FACTOR4 | * | CW | 40.7 | 36.1 - 40.8 | 12 | OAR26_40722597.1 | s45791.1 | 0.15 | 0.355 | 2.87 | 0.13 | -0.139 | 0.39 |

^1^: *, p < 5%; **, p < 1%; ***, p < 0.1%. ^2^: position of a second significant QTL indicated between parentheses. ^3^: SNPs flanking the haplotype with significant association. ^4^: Percentage of phenotypic variance explained by haplotype. CW, Chromosome Wide; GW, Genome Wide; No haplotypes, Number of haplotypes; Freq, Haplotype frequency
