# Supplementary material for: Identification of QTLs for behavioral reactivity to social separation and humans in sheep using the OvineSNP50 BeadChip
Source: BMC Genomics. 2014 Sep 9;15(1):778. doi: 10.1186/1471-2164-15-778 (PMC4171556; doi:10.1186/1471-2164-15-778)
Supplement: Supplementary file 5 — Additional file 5: Table S3: Complete list of QTLs detected by LDLA analysis. This file contains a table that lists all the significant QTLs found by joint linkage and association analysis for the 16 traits and provides the significance, position of maximum likelihood ratio test, confidence interval, number of haplotypes and the name of flanking markers. (DOCX 24 KB) [file 12864_2014_6464_MOESM5_ESM.docx]

**Additional file 5: Table S3** Complete list of QTL detected in LDLA analysis.

| **OAR** | **Trait** | **Significance^1^** | **Pos^2^ (MB)** | **No. haplotypes** | **Flanking markers^3^** | |
| --- | --- | --- | --- | --- | --- | --- |
| 1 | AT2_LBLEAT | ** | 175.9 | 8 | OAR1_175699560.1 | OAR1_175957082.1 |
| 1 | AT2_PROX | *** | 261.4 | 10 | OAR1_261353918.1 | OAR1_261426539.1 |
| 1 | CT1_LOCOM | ** | 30.7 | 10 | OAR1_30673378.1 | s11621.1 |
| 1 | CT2_SEEN | *** | 87.1 | 12 | s21838.1 | OAR1_87136648.1 |
| 1 | IBT_HBLEAT | *** | 265.1 | 11 | OAR1_265023775.1 | OAR1_265099083.1 |
| 1 | ISO_HBLEAT | ** | 265.1 | 11 | OAR1_265023775.1 | OAR1_265099083.1 |
| 2 | AT1_HBLEAT | *** | 246.6 | 11 | OAR2_246577997.1 | s51975.1 |
| 2 | AT1_LBLEAT | ** | 204.7 | 12 | OAR2_204684212.1 | OAR2_204712172.1 |
| 2 | AT2_HBLEAT | ** | 219.1 | 11 | OAR2_219089298.1 | OAR2_219181654.1 |
| 2 | AT2_LBLEAT | ** | 164.6 | 8 | OAR2_164592801.1 | OAR2_164635032.1 |
| 2 | AT2_LOCOM | ** | 181.7 | 11 | OAR2_181662730.1 | OAR2_181764446.1 |
| 2 | IBT_LOCOM | *** | 14.8 | 13 | OAR2_14765360.1 | OAR2_14833429.1 |
| 2 | ISO_HBLEAT | ** | 143.8 | 11 | s01640.1 | OAR2_143893183.1 |
| 2 | ISO_LBLEAT | ** | 204.7 | 12 | OAR2_204684212.1 | OAR2_204712172.1 |
| 2 | ISO_LOCOM | ** | 111.6 | 13 | s36949.1 | s61069.1 |
| 3 | AT1_LOCOM | ** | 180.2 | 11 | OAR3_180174414.1 | OAR3_180275424.1 |
| 3 | AT2_HBLEAT | ** | 186.5 | 11 | s15055.1 | OAR3_186598528.1 |
| 3 | AT2_LBLEAT | *** | 132.4 | 9 | OAR3_132393813.1 | OAR3_132452051.1 |
| 3 | CT1_LBLEAT | ** | 1.0 | 14 | s72862.1 | s71636.1 |
| 3 | ISO_LBLEAT | ** | 3.6 | 14 | OAR3_3419701.1 | s03864.1 |
| 4 | AT2_LOCOM | *** | 44.7 | 13 | OAR4_44642698.1 | OAR4_44700812.1 |
| 4 | AT2_PROX | ** | 68.7 | 12 | OAR4_68678863.1 | OAR4_68712576.1 |
| 4 | CT2_DIST | ** | 27.8 | 13 | OAR4_27722499.1 | OAR4_27818517.1 |
| 5 | AT1_LBLEAT | ** | 80.3 | 10 | OAR5_80246247.1 | OAR5_80304302.1 |
| 5 | AT2_HBLEAT | ** | 80.3 | 10 | OAR5_80246247.1 | OAR5_80304302.1 |
| 5 | AT2_LOCOM | ** | 49.2 | 12 | OAR5_49181962.1 | OAR5_49283556.1 |
| 5 | CT1_HBLEAT | *** | 92.1 | 11 | OAR5_92064206.1 | OAR5_92169254.1 |
| 5 | IBT_HBLEAT | ** | 47.8 | 14 | OAR5_47774570.1 | s12940.1 |
| 5 | ISO_HBLEAT | *** | 92.1 | 11 | OAR5_92064206.1 | OAR5_92169254.1 |
| 6 | AT1_HBLEAT | ** | 106.9 | 11 | s05095.1 | s25229.1 |
| 6 | ISO_HBLEAT | ** | 106.9 | 11 | s05095.1 | s25229.1 |
| 7 | AT1_HBLEAT | ** | 18.3 | 14 | s40821.1 | OAR7_18356443.1 |
| 7 | AT1_LBLEAT | ** | 52.1 | 9 | OAR7_52124140.1 | OAR7_52157129.1 |
| 7 | AT1_LOCOM | ** | 81.9 | 9 | OAR7_81929691.1 | OAR7_81975578.1 |
| 7 | CT2_DIST | ** | 7.8 | 12 | OAR7_7821604.1 | s56405.1 |
| 7 | ISO_HBLEAT | ** | 18.3 | 14 | s40821.1 | OAR7_18356443.1 |
| 7 | ISO_LBLEAT | ** | 52.1 | 9 | OAR7_52124140.1 | OAR7_52157129.1 |
| 8 | AT1_LBLEAT | ** | 69.8 | 12 | OAR8_69819161.1 | OAR8_69854114.1 |
| 8 | AT2_LOCOM | ** | 6.6 | 12 | OAR8_6595131.1 | OAR8_6643214.1 |
| 8 | ISO_LOCOM | *** | 35.3 | 11 | s02853.1 | OAR8_35349389.1 |
| 9 | AT2_HBLEAT | ** | 84.3 | 10 | s65296.1 | OAR9_84346672.1 |
| 9 | AT2_LOCOM | ** | 92.3 | 11 | OAR9_92200938.1 | OAR9_92302903.1 |
| 9 | CT1_LBLEAT | ** | 65.7 | 14 | OAR9_65626804.1 | OAR9_65660326.1 |
| 9 | ISO_HBLEAT | ** | 43.7 | 13 | OAR9_43640216_X.1 | OAR9_43692772.1 |
| 10 | CT2_DIST | ** | 54.3 | 12 | OAR10_54264286.1 | OAR10_54347258.1 |
| 10 | ISO_HBLEAT | ** | 47.2 | 11 | OAR10_47209581.1 | OAR10_47249054.1 |
| 11 | AT1_LBLEAT | ** | 45.3 | 11 | OAR11_45273342.1 | s70451.1 |
| 11 | ISO_LBLEAT | ** | 35.8 | 12 | OAR11_35758049.1 | OAR11_35836726.1 |
| 12 | AT1_LBLEAT | *** | 58.6 | 9 | DU490596_503.1 | OAR12_58655930.1 |
| 12 | AT2_LBLEAT | *** | 56.3 | 13 | s63508.1 | OAR12_56302501_X.1 |
| 12 | CT1_LBLEAT | ** | 45.0 | 10 | s23222.1 | s68281.1 |
| 12 | CT2_DIST | ** | 32.6 | 10 | OAR12_32559849.1 | OAR12_32617508.1 |
| 12 | CT2_SEEN | ** | 39.7 | 10 | OAR12_39622345.1 | OAR12_39680259.1 |
| 12 | ISO_HBLEAT | ** | 39.4 | 12 | OAR12_39336634.1 | s19292.1 |
| 12 | ISO_LBLEAT | *** | 56.2 | 13 | s63508.1 | OAR12_56302501_X.1 |
| 13 | AT1_LBLEAT | ** | 64.5 | 9 | s65913.1 | OAR13_64562058.1 |
| 13 | AT2_HBLEAT | *** | 31.9 | 10 | OAR13_31877042.1 | OAR13_31918462.1 |
| 13 | CT1_HBLEAT | *** | 45.6 | 13 | s73104.1 | s43103.1 |
| 13 | ISO_HBLEAT | *** | 34.0 | 10 | OAR13_33983722.1 | OAR13_34056956.1 |
| 13 | ISO_LBLEAT | ** | 64.5 | 9 | s65913.1 | OAR13_64562058.1 |
| 15 | AT1_LBLEAT | ** | 74.7 | 9 | OAR15_74672514.1 | OAR15_74759937.1 |
| 16 | AT1_HBLEAT | *** | 44.4 | 11 | s23014.1 | s48431.1 |
| 16 | AT2_HBLEAT | *** | 42.8 | 10 | OAR16_42806888.1 | OAR16_42993224.1 |
| 16 | AT2_LBLEAT | ** | 30.1 | 9 | OAR16_30048572.1 | OAR16_30230297.1 |
| 16 | IBT_HBLEAT | ** | 61.9 | 13 | OAR16_61906879.1 | OAR16_61967916.1 |
| 16 | ISO_HBLEAT | *** | 44.4 | 11 | s23014.1 | s48431.1 |
| 17 | AT1_HBLEAT | ** | 12.7 | 12 | s42157.1 | OAR17_12809597.1 |
| 17 | CT1_HBLEAT | ** | 43.6 | 12 | s00294.1 | OAR17_43682916.1 |
| 17 | CT2_DIST | ** | 11.1 | 11 | OAR17_11099220.1 | DU324670_456.1 |
| 17 | IBT_HBLEAT | ** | 12.7 | 12 | s42157.1 | OAR17_12809597.1 |
| 17 | ISO_HBLEAT | *** | 12.7 | 12 | s42157.1 | OAR17_12809597.1 |
| 17 | ISO_LBLEAT | *** | 33.9 | 9 | OAR17_33932204.1 | OAR17_33976578.1 |
| 18 | AT2_HBLEAT | *** | 45.2 | 14 | OAR18_45139986.1 | s59000.1 |
| 19 | AT2_HBLEAT | ** | 51.3 | 9 | OAR19_51269847.1 | OAR19_51317745.1 |
| 19 | AT2_LOCOM | *** | 18.9 | 9 | OAR19_18888791.1 | s24963.1 |
| 19 | CT2_DIST | ** | 33.6 | 13 | OAR19_33605872.1 | s29797.1 |
| 19 | IBT_LOCOM | ** | 18.0 | 10 | s56027.1 | OAR19_18010247.1 |
| 20 | AT2_HBLEAT | *** | 29.0 | 9 | OAR20_28937911.1 | OAR20_29051859.1 |
| 20 | AT2_LBLEAT | *** | 17.2 | 14 | OAR20_17174254.1 | OAR20_17276357.1 |
| 20 | CT2_SEEN | ** | 7.7 | 10 | OAR20_7626319.1 | OAR20_7794638.1 |
| 20 | IBT_LOCOM | *** | 40.5 | 15 | OAR20_40492055.1 | s25588.1 |
| 21 | AT1_LBLEAT | *** | 7.2 | 14 | s54902.1 | OAR21_7730122.1 |
| 21 | AT1_VIGIL | ** | 13.2 | 15 | OAR21_13212843.1 | OAR21_13268403.1 |
| 21 | AT2_HBLEAT | ** | 37.8 | 12 | s33769.1 | s59207.1 |
| 21 | IBT_LOCOM | ** | 23.7 | 10 | OAR21_23698875.1 | OAR21_23776540.1 |
| 21 | ISO_LBLEAT | *** | 38.8 | 8 | s17503.1 | s68333.1 |
| 22 | AT1_HBLEAT | ** | 34.6 | 13 | OAR22_34620231.1 | s71494.1 |
| 23 | AT2_LOCOM | ** | 54.1 | 10 | OAR23_54064411.1 | s31567.1 |
| 23 | HUMAPPRO | ** | 32.2 | 12 | OAR23_32137172.1 | s55273.1 |
| 23 | IBT_LOCOM | ** | 61.9 | 8 | s30087.1 | OAR23_61932991.1 |
| 24 | AT1_HBLEAT | ** | 7.0 | 13 | s54640.1 | s61820.1 |
| 24 | AT1_LOCOM | ** | 24.0 | 13 | OAR24_23977454.1 | OAR24_24088024.1 |
| 24 | CT2_DIST | ** | 8.3 | 10 | s56297.1 | OAR24_8402585.1 |
| 24 | ISO_LBLEAT | ** | 8.1 | 11 | s04466.1 | s56297.1 |
| 26 | AT1_HBLEAT | ** | 13.9 | 12 | OAR26_13918913.1 | OAR26_14049024.1 |
| 26 | CT2_DIST | ** | 5.1 | 11 | OAR26_5062133.1 | DU381007_457.1 |
| 26 | IBT_HBLEAT | ** | 45.4 | 13 | s31164.1 | s16475.1 |
| 26 | ISO_HBLEAT | ** | 45.2 | 15 | s65487.1 | OAR26_45256559.1 |

^1^: **, 1% genome wise threshold; ***, 0.1% genome wise threshold. ^2^: QTL position given in Mb . ^3^: SNPs flanking the haplotype with significant association. Only QTL reaching the 1% genome-wise significant p-value were listed in the table.
