# Supplementary material for: Identification of QTLs for behavioral reactivity to social separation and humans in sheep using the OvineSNP50 BeadChip
Source: BMC Genomics. 2014 Sep 9;15(1):778. doi: 10.1186/1471-2164-15-778 (PMC4171556; doi:10.1186/1471-2164-15-778)
Supplement: Supplementary file 6 — Additional file 6: Table S5: Estimates of heritabilities and variances ± S.E for behavioral and physiological traits. This file contains heritability of traits analyzed in this study, proportion of phenotypic variance attributed to maternal, litter and residual effects and the total phenotypic variance for each trait. (DOCX 18 KB) [file 12864_2014_6464_MOESM6_ESM.docx]

Additional file 6: Table S5. Estimates of heritabilities and variances ± S.E for behavioral and physiological traits

|  |  | Component | | | |  |
| --- | --- | --- | --- | --- | --- | --- |
|  | n | Animal (h^2^) | Dam (m^2^) | Litter (c^2^) | Residual (e^2^) | Total (σ_p_^2^) |
| AT1-LOCOM  AT2-LOCOM  CT1-LOCOM  IBT-LOCOM  AT1-HBLEAT  AT2-HBLEAT  CT1-HBLEAT  IBT-HBLEAT  AT1-LBLEAT  AT2-LBLEAT  CT1-LBLEAT  AT1-VIGIL  AT2-PROX  CT2-DIST  CT2-SEEN  CORT  ISO-LOCOM  ISO-HBLEAT  ISO-LBLEAT  HUMAPPRO  FACTOR1  FACTOR2  FACTOR3  FACTOR4 | 1066  1066  1099  1094  1099  1099  1099  1094  1099  1099  1099  1066  1033  1099  1099  477  1062  1094  1099  1099  1029  1029  1029  1029 | 0.23 ± 0.07  0.20 ± 0.08  0.12 ± 0.06  0.21 ± 0.07  0.35 ± 0.06  0.35 ± 0.08  0.40 ± 0.07  0.37 ± 0.08  0.45 ± 0.09  0.27 ± 0.06  0.21 ± 0.12  0.21 ± 0.09  0.17 ± 0.05  0.20 ± 0.06  0.17 ± 0.06  0.16 ± 0.07  0.29 ± 0.08  0.51 ± 0.08  0.43 ± 0.09  0.21 ± 0.06  0.49 ± 0.08  0.26 ± 0.08  0.33 ± 0.08  0.36 ± 0.10 | 0.00 ± 0.0  0.00 ± 0.0  0.02 ± 0.03  0.00 ± 0.0  0.00 ± 0.0  0.00 ± 0.0  0.00 ± 0.0  0.03 ± 0.04  0.02 ± 0.03  0.00 ± 0.0  0.02 ± 0.04  0.04 ± 0.04  0.00 ± 0.0  0.00 ± 0.0  0.00 ± 0.0  0.00 ± 0.0  0.00 ± 0.0  0.01 ± 0.03  0.02 ± 0.04  0.00 ± 0.0  0.00 ± 0.0  0.00 ± 0.0  0.00 ± 0.0  0.00 ± 0.0 | 0.00 ± 0.0  0.00 ± 0.0  0.08 ± 0.05  0.00 ± 0.0  0.03 ± 0.04  0.06 ± 0.04  0.07 ± 0.04  0.04 ± 0.05  0.03 ± 0.05  0.00 ± 0.0  0.00 ± 0.0  0.07 ± 0.05  0.05 ± 0.05  0.00 ± 0.0  0.00 ± 0.0  0.00 ± 0.0  0.00 ± 0.0  0.04 ± 0.05  0.01 ± 0.05  0.00 ± 0.0  0.04 ± 0.05  0.00 ± 0.0  0.00 ± 0.0  0.00 ± 0.0 | 0.76 ± 0.07  0.79 ± 0.08  0.78 ± 0.07  0.78 ± 0.07  0.62 ± 0.07  0.58 ± 0.08  0.52 ± 0.08  0.55 ± 0.08  0.50 ± 0.08  0.73 ± 0.06  0.77 ± 0.11  0.66 ± 0.04  0.77 ± 0.07  0.80 ± 0.07  0.82 ± 0.07  0.83 ± 0.07  0.70 ± 0.08  0.44 ± 0.07  0.53 ± 0.08  0.79 ± 0.07  0.45 ± 0.08  0.74 ± 0.08  0.66 ± 0.08  0.64 ± 0.10 | 75.13 ± 0.26  17.49 ± 0.13  7.90 ± 0.09  2.43 ± 0.05  1.69 ± 0.04  1.33 ± 0.03  1.23 ± 0.03  1.34 ± 0.03  0.14 ± 0.01  0.09 ± 0.00  0.09 ± 0.00  88.70 ± 0.28  252.1 ± 0.49  1.32 ± 0.03  48.04 ± 0.20  0.03 ± 0.00  1.40 ± 0.04  2.02 ± 0.04  1.60 ± 0.04  1.68 ± 0.04  0.95 ± 0.03  0.91 ± 0.03  0.98 ± 0.03  1.12 ± 0.03 |

h^2^, m^2^, c^2^, e^2^ = proportion of phenotypic variance attributed to additive genetic, maternal, litter and residual effects, respectively; σ_p_^2^ = phenotypic variance.
